# Enzymatic DNA Reaction Networks for Orchestrating Stimuli‐Dependent Temporal Molecular Pulse

**DOI:** 10.1002/advs.202520984

**Published:** 2026-04-20

**Authors:** Jiayu Yang, Yali Chang, Zibin Chu, Linghao Zhang, Tengfang Zhang, Dai Erhei, Xin Su, Zhe Yin

**Affiliations:** ^1^ State Key Laboratory of Organic‐Inorganic Composites, Beijing Key Laboratory of Bioprocess, Beijing Advanced Innovation Center for Soft Matter Science and Engineering, College of Life Science and Technology Beijing University of Chemical Technology Beijing China; ^2^ Hebei Key Laboratory of Immune Mechanism of Major Infectious Diseases and New Technology of Diagnosis and Treatment The Fifth Hospital of Shijiazhuang Hebei Medical University Shijiazhuang China; ^3^ State Key Laboratory of Pathogen and Biosecurity Academy of Military Medical Sciences Beijing China

**Keywords:** DNA circuits, DNA nanotechnology, Molecular diagnostics, Nuclease, Temporal signal

## Abstract

Temporal dynamics are a hallmark of cellular information processing, yet cell‐free biosensors still mainly rely on amplitude‐based fluorescence readouts that face multiplex limits from limited spectral channels, cross‐talk, and rising complexity. Here we present an enzymatic DNA reaction network (EDRN) that introduces time as an orthogonal coding dimension by translating stimuli into programmable temporal pulses. EDRN integrates a polymerase‐based concentration converter that normalizes inputs into a standardized universal strand (Us) at set doses with an exonuclease‐driven temporal decoder that converts Us dose into transient fluorescence pulses. This modular separation provides orthogonal enzymatic control over pulse amplitude and lifetime, enabling a wide programmable range without delicate structure‐dependent fine‐tuning. By tuning Us production through single‐ or double‐layer converters, pulse lifetimes can be programmed from ∼10 min to ∼5 h and temporal signatures assigned across targets. As a proof of concept, we demonstrate multiplex bacterial nucleic‐acid detection in one tube, where targets are resolved by time‐color encoding, achieving ten‐plex readout using four fluorophores with multiple temporal windows. Clinical validation on 32 specimens (22 positives and 10 healthy controls) showed consistency with sequencing. These results establish a general stimulus‐to‐time strategy for nucleic‐acid circuits and expand the multiplexing capacity of fluorescence‐based cell‐free biosensing.

## Graphical abstract

We present an enzymatic DNA reaction network (EDRN) that encodes nucleic‐acid targets in time, converting inputs into a universal strand and then into programmable transient fluorescence pulses. With time‐color multiplexing, EDRN enables single‐tube high‐plex nucleic acid detection and shows strong agreement with clinical sequencing across 32 specimens.

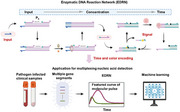

## 1 Introduction

Cell‐free biosensors have emerged as a versatile platform for molecular detection and information processing because they offer high programmability and modularity while avoiding the regulatory and compositional complexity of living systems [1, 2, 3]. By reconstituting defined biochemical reactions in vitro, these systems enable precise control over sensing pathways and have been widely explored for clinical diagnostics, environmental monitoring, and synthetic biology [4]. Most existing cell‐free biosensors translate molecular recognition events‐such as nucleic‐acid hybridization, enzyme activation, or strand‐displacement reactions‐into changes in fluorescence signals [5, 6, 7]. However, when multiplex recognition is required, limited optical channel capacity and spectral cross‐talk intersect with the increasing molecular complexity of one‐pot mixtures (more probes/primers/strands) [8, 9, 10], which elevates the likelihood of off‐target interactions and target‐dependent response bias, often necessitating extensive empirical optimization or splitting panels into multiple reactions‐thereby eroding the simplicity, throughput, and scalability that make cell‐free biosensors attractive [11, 12].

**FIGURE 1:**
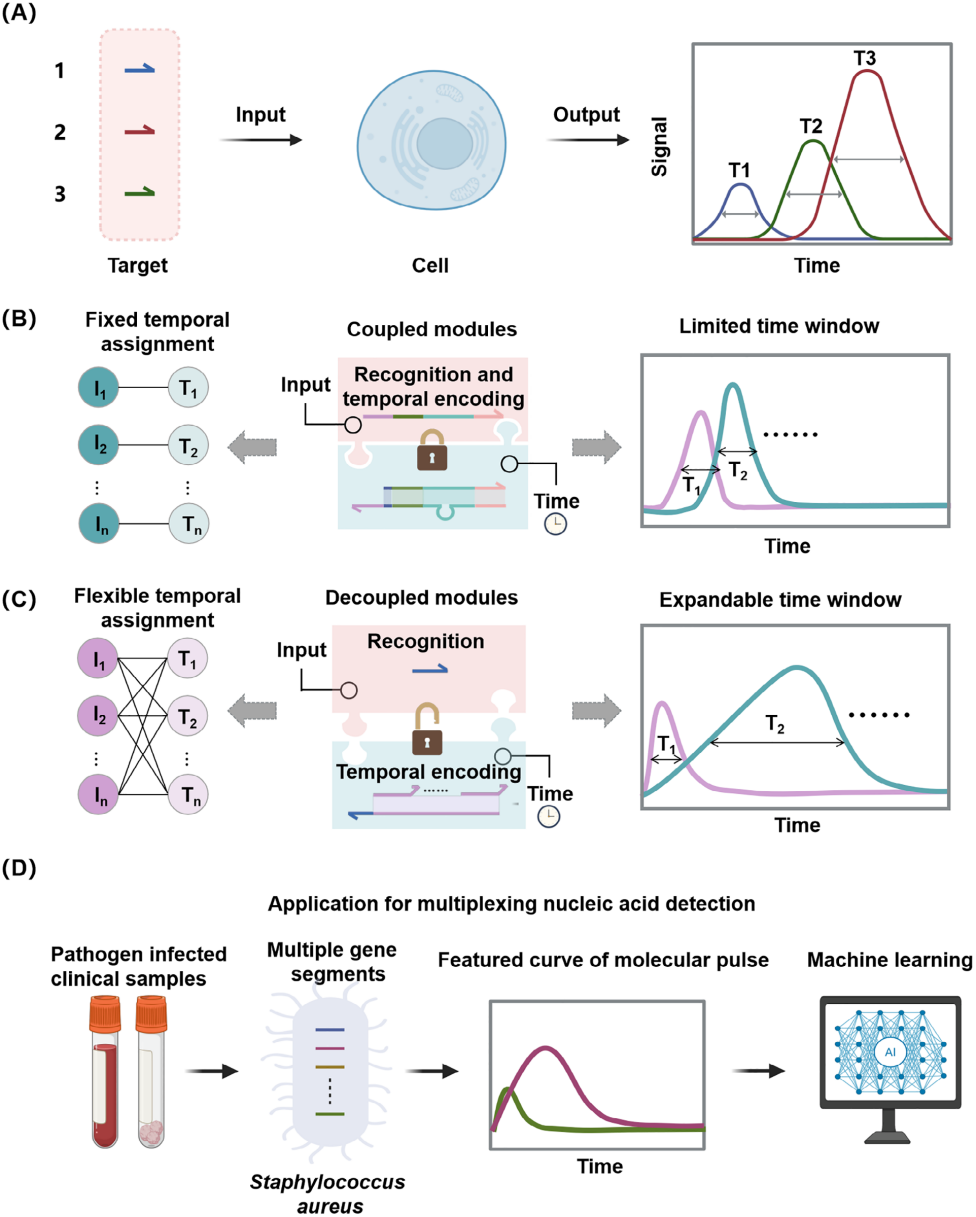
Enzymatic DNA reaction network (EDRN) for stimulus‐to‐time encoding. (A) Cell responds diverse molecular inputs into time resolved molecular signals. (B) Cell free biosensors based on conventional DNA reaction networks typically couple input recognition and temporal encoding within the same molecular module. As a result, each input is restricted to a predefined temporal response, and the temporal window is difficult to expand. (C) EDRN decouples input recognition from temporal encoding. A polymerase‐based concentration converter maps diverse inputs to a standardized intermediate, which is subsequently interpreted by a temporal decoder to generate programmable molecular pulses. This modular architecture enables flexible mapping between stimulus identity and temporal behavior, as well as expansion of the temporal window. (D) Multiplex detection of bacterial genes using EDRN. Distinct genetic targets generate non‐overlapping temporal signatures within a single fluorophore channel. Coupling temporal encoding with four fluorophores expands the assay to 10 targets per tube, and machine learning‐based analysis of pulse features enables accurate clinical molecular diagnostics.

Cells routinely translate diverse stimuli into time‐dependent molecular signals, where when and for how long a signal is produced can be as informative as how strong it is [13, 14, 15]. For example, signaling pathways can encode stimulus identity or strength through distinctive temporal patterns such as delayed activation [16], transient pulses [17], sustained plateaus, or oscillations [18], arising from multilayer enzymatic cascades [19], feedback regulation [20], and controlled degradation (Figure 1A) [21]. If cell‐free biosensors could emulate such temporal signaling in a programmable manner, temporal features (e.g., onset time, pulse width, and decay trajectory) could serve as an additional and orthogonal coding dimension [22, 23, 24, 25]. In principle, combining temporal encoding with the established advantage of fluorophore diversity would greatly expand multiplexing capacity: multiple targets could be resolved within a single spectral channel by distinct time signatures, and the same temporal windowing strategy could be applied across several colors to further multiply the number of resolvable analytes in one pot [26, 27]. Dynamic DNA nanotechnology provides a compelling foundation for implementing time‐resolved behaviors in vitro, and a range of nucleic‐acid reaction networks have been developed to generate non‐equilibrium dynamics [28, 29, 30], including delayed responses, transient pulses, and programmable degradation [31]. However, scalable temporal programming for multiplex sensing remains difficult in practice [32, 33]. In some nucleic‐acid circuits, target recognition is tightly coupled to system kinetics because temporal behavior is governed by sequence‐ and structure‐dependent parameters such as toehold length, binding energies, and secondary structures (Figure 1B) [34, 35]. As a result, a given input at a given concentration often yields a largely fixed temporal output, and reassigning or expanding temporal signatures across different targets typically requires extensive sequence redesign and delicate kinetic fine‐tuning. These constraints complicate panel expansion, reduce portability across targets, and ultimately limit the use of temporal dynamics as a general‐purpose encoding layer for multiplex cell‐free biosensing‐motivating the need for a modular framework that decouples stimulus recognition from temporal programming.

Here, we introduce a cell‐free biosensing strategy based on a synthetic enzymatic DNA reaction network (EDRN) that decouples stimulus recognition from temporal programming, thereby enabling molecular identity and temporal behavior to be tuned independently (Figure 1C). Therefore, temporal signatures can be freely and predictably assigned to stimuli, and the temporal window can be easily expanded. In this design, a polymerase‐based concentration converter transforms diverse input strands into a standardized universal strand (Us) with certain amount, which is then interpreted by an exonuclease‐driven temporal decoder to generate transient molecular pulses. This modular separation acts as orthogonal enzymatic levers, enabling robust and predictable control over both pulse amplitude and lifetime. Critically, the amount of Us generated by the polymerase determines pulse duration, establishing concentration as the key variable linking molecular identity to time. By tuning Us production through single‐ or double‐layer concentration converters, pulse lifetimes can be programmed across a wide dynamic range‐from 10 min to 5 h‐without reliance on delicate DNA structure‐based kinetic adjustments. The system supports the full spectrum of encoding modes: the same input yielding different temporal behaviors, different inputs producing distinct behaviors, and different inputs converging on a shared behavior.

As a proof of concept, we exploited EDRN for multiplexed bacterial nucleic acid detection (Figure 1D). Distinct genetic targets produced non‐overlapping temporal signatures, enabling a single fluorophore channel to encode multiple inputs. By coupling the strategy with four commonly used fluorophores, we extended the multiplexing capacity to 10 targets within a single‐tube assay. Integration with machine learning‐based analysis further enhanced classification performance, with the rich temporal features of the pulse signals yielding high accuracy in synthetic samples. In a cohort of 32 patient samples (22 positive cases and 10 healthy controls), the detection outcomes exhibited high consistency with sequencing results. Beyond diagnostics, this stimulus‐to‐time encoding paradigm establishes a versatile principle for nucleic acid circuits, advancing opportunities in dynamic biosensing, adaptive therapeutics, and programmable synthetic biological regulation.

## 2 Results and Discussion

### 2.1 Independent Operation of Concentration Converter and Temporal Decoder

**FIGURE 2:**
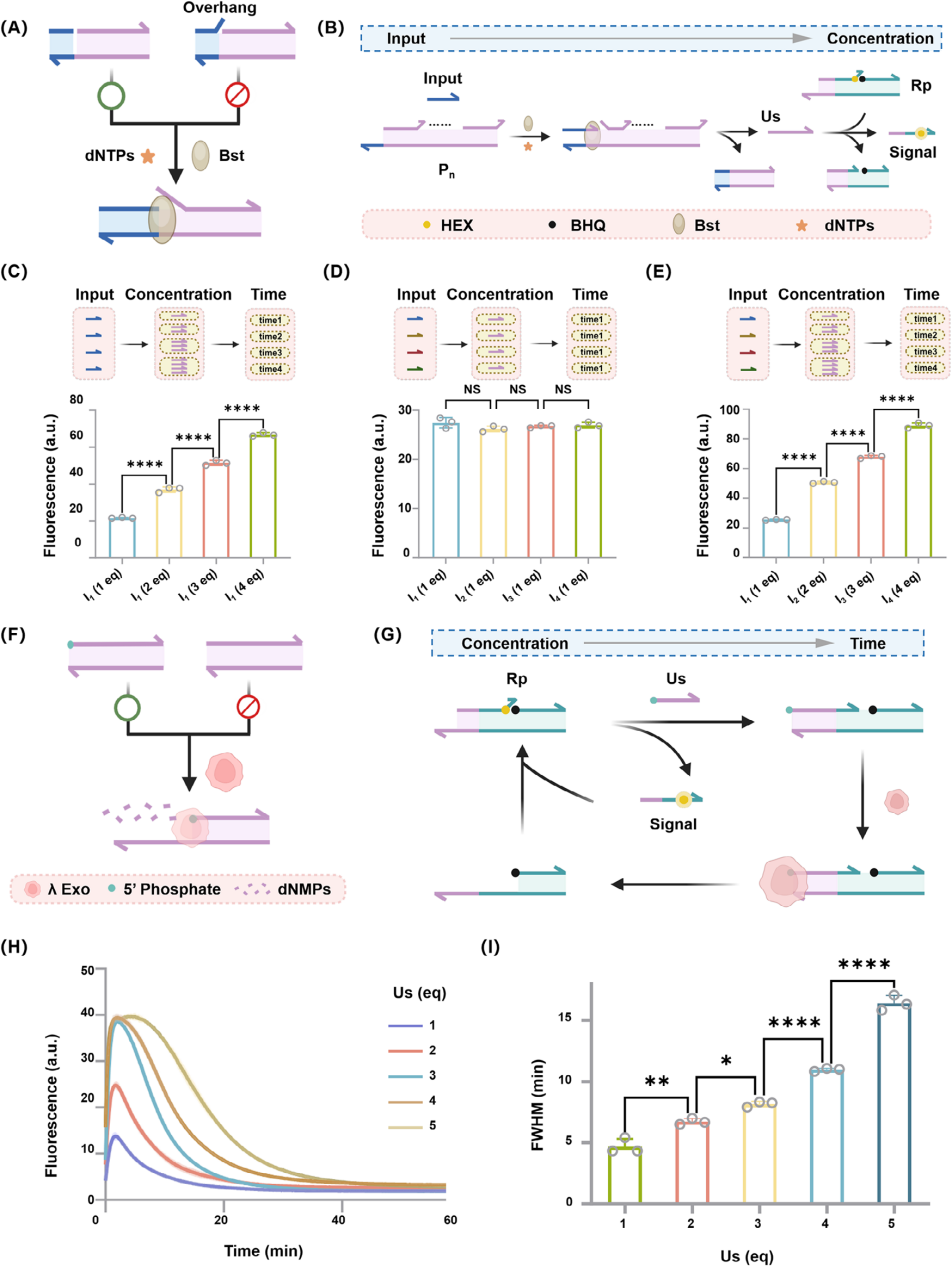
Independent operation of the concentration converter and temporal decoder modules. (A) The overhang of primer strand can inhibit the extension of Bst. (B) Schematic illustration of the concentration converter. Upon hybridization of the input with P_n_, Bst mediates the generation of the Us, successfully accomplishing concentration conversion. (C) Data extracted from Figure S4B. A positive correlation was observed between fluorescence endpoint values and Us concentration. (D) Data extracted from Figure S5B. Multiple distinct inputs converge to the same concentration of Us. (E) Data extracted from Figure S6B. Multiple distinct inputs generated different concentrations of Us. (F) Enzymatic activity of λ Exo. λ Exo recognizes double‐stranded DNA with a 5′‐phosphate and catalyzes processive degradation to release dNMPs, whereas strands with a free 5′‐hydroxyl end remain resistant to cleavage. (G) Schematic illustration of the temporal decoder. The concentration of Us is translated into temporal output, whereby higher concentrations extend the signal duration, whereas lower concentrations shorten it. (H) Fluorescence curves of the temporal decoder with different Us concentration. (I) FWHM extracted from panel G. FWHM of the temporal decoder increased proportionally with rising Us concentrations. Experiments in panels B‐D were performed with 200 nM Rp, 50 nM input, 50 nM P_n_ and 160 U/mL Bst. Experiments in panel G were performed with 100 nM Rp and 5.5 U/mL λ Exo. All experiments were performed in 1× Lambda reaction buffer at 25°C, and data are presented as mean ± SD (n = 3 independent experiments).

To establish EDRN, we first delineated its two modules‐the Concentration Converter and the Temporal Decoder. Input strands trigger the circuit, and a strand‐displacement reporter provides quantitative fluorescence readouts of processed states. To suppress spurious polymerase extension in the absence of input, the 3′ end of Us was capped with a 5‐nt T overhang (Figure 2A). In the Concentration Converter, an input hybridizes to its probe and, via Bst‐mediated extension and strand displacement, releases Us (Figure 2B). The released Us is then read out by a reporter in which Us displaces a quenched signal strand to generate fluorescence; no signal appears without input (Figure S1A,B). To ensure robust performance at ambient temperature, we confirmed that Bst polymerase retains similar activity across 25–65°C (Figure S2A‐C). Because Bst polymerase supports consecutive strand displacement, Us yield is tunable, enabling identical inputs to be converted into distinct concentration states. Moreover, sequencing showed that Bst extension products matched the designed sequence, with no detectable errors (Figure S3). We defined 50 nM Us as one equivalent (eq); under different conditions, 50 nM input produced 1–4 eq (Figure S4A‐B). Conversely, distinct inputs could be programmed either to converge on the same concentration (Figure S5A,B) or to diverge into unique concentration profiles (Figure S6A,B). Bulk fluorescence measurements confirmed that Us production maps to the designated inputs (Figure 2C‐E), and gel electrophoresis validated the conversions: lanes 7–10 showed increasing band intensity with higher Us, consistent with a positive correlation between concentration and grey level (Figure S7A,B). Together, these results establish Us as a unifying intermediate that decouples input recognition from downstream temporal processing.

We next interrogated the Temporal Decoder, which converts Us concentration into time‐encoded fluorescence via λ‐exonuclease‐mediated degradation. λ exonuclease (λ Exo) recognizes the 5′‐phosphorylated end of duplex DNA and degrades the phosphorylated strand in the 5′→3′ direction; removing the 5′ phosphate markedly reduces activity (Figure 2F). A 5′‐phosphorylated Us first invades the reporter duplex, displacing the quenched Signal strand and turning fluorescence on. As λ Exo digests Us, the binding site reopens and the Signal strand re‐hybridizes to its quencher complement, generating pulse‐like signal (Figure 2G). No signal was found in the absence of input (Figure S8A,B). Varying the amount of Us deterministically tuned the kinetics (Figure 2H). Systematic titration from 1 to 5 equivalents of Us broadened the full width at half maximum (FWHM) a 3.2‐fold increase (Figure 2I). Gel analyses corroborated the underlying concentration‐to‐time conversion (Figure S9).

Together, these data establish two independent modules the concentration converter and the temporal decoder. These two modules can be connected by Us: input is first encoded as a Us concentration and then translated into a temporal signature. The framework supports multiple encoding modes—(i) the same input yielding different temporal behaviors, (ii) different inputs yielding distinct behaviors, and (iii) different inputs converging on the same behavior—thereby positioning Us as a central intermediate and time as a tunable output for higher‐order temporal control in synthetic DNA networks.

### 2.2 Integration of the Concentration Converter and Temporal Decoder Modules for Programming Molecular Pulses

**FIGURE 3:**
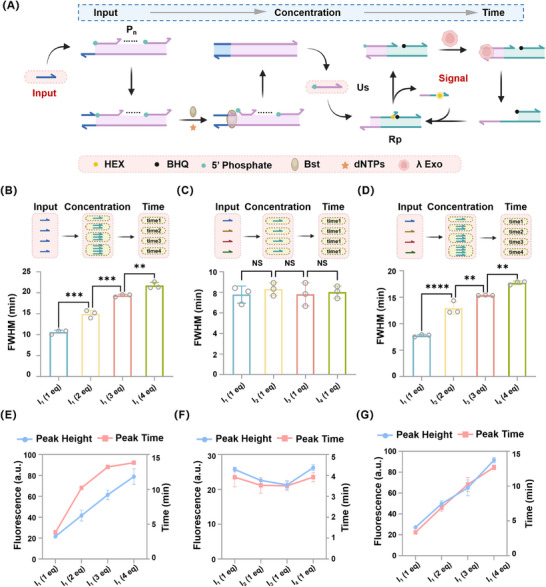
Integration of the concentration converter and temporal decoder modules for programming molecular pulses. (A) Schematic diagram of concentration‐to‐time conversion. Molecular inputs are processed by the concentration converter to generate Us, which is then interpreted by the temporal decoder to produce rise‐fall pulse trajectories. (B) FWHM extracted from Figure S11B. Identical inputs yield distinct temporal signatures when different amounts of Us are produced, resulting in divergence of FWHM. (C) FWHM extracted from Figure S12B. Distinct inputs can converge on the same temporal output, producing overlapping trajectories and nearly indistinguishable extracted features. (D) FWHM extracted from Figure S13B. Different inputs can be resolved into distinct temporal windows, yielding separated pulses characterized by clear differences in FWHM. (E) Peak height and time extracted from Figure S11B. Both variables increase with rising Us concentration. (F) Peak height and time extracted from Figure S12B. The small changes observed in both variables as Us concentration increased demonstrate that distinct inputs were successfully transformed into identical temporal characteristics. (G) Peak height and time extracted from Figure S13B. Both variables are positively correlated with Us concentration. Experiments in B‐D were performed with 200 nM Rp, 50 nM I_n_, 50 nM P_n_, 5.5 U/mL λ Exo and 160 U/mL Bst in 1× Lambda reaction buffer at 25°C. Data are presented as mean ± SD (n = 3 independent experiments).

To test end‐to‐end programmability, we linked the polymerase‐based Concentration Converter to the λ‐exonuclease Temporal Decoder (EDRN) (Figure 3A). An input strand first hybridizes to Concentration Converter and, under Bst‐mediated extension with strand displacement, liberates Us. Us then engages the toehold of Temporal Decoder, displacing the quenched Signal strand to turn fluorescence on; subsequent λ Exo digestion of Us restores the vacant site, allowing Signal to re‐hybridize and quench—yielding a transient rise‐fall pulse. Thus, the pathway implements a deterministic mapping from molecular identity to temporal signature. Functionally, the two modules act as orthogonal enzymatic controls: Bst sets the yield of Us (thereby specifying the programmed dose delivered to the decoder), while λ Exo sets the removal rate of Us (thereby fixing the pulse lifetime/width). In practice, the amount of Us produced by the converter defines pulse duration, establishing concentration as the quantitative link between input identity and time.

We next characterized stimulus‐dependent temporal sensing. First, for a given input held at a fixed concentration, tuning the converter to yield 1–4 eq of Us produced proportionally longer pulses, with FWHM expanding from 10 to 22 min (Figure S10A,B). Both peak intensity and time‐to‐peak increased, demonstrating that a single input can be programmed to produce distinct temporal signatures by adjusting Us yield (Figure 3B,E). Second, when different inputs were each assigned to 1 eq of Us, their temporal trajectories overlapped without variation in peak amplitude or time‐to‐peak (Figure 3C,F; Figure S11A,B). Third, when distinct inputs were each converted across 1–4 eq, the resulting pulses separated in time and amplitude: FWHM broadened from 8 to 16 min with concomitant increases in peak intensity and time‐to‐peak, enabling discrimination by non‐overlapping temporal signatures (Figure 3D,G; Figure S12A,B). The integration of Concentration Converter and Temporal Decoder was confirmed by gel electrophoresis (Figure S13). We then probed enzyme levels as independent control knobs. Increasing Bst accelerated Us production and shortened pulses, reducing FWHM from 30 to 9 min, with coordinated shifts in peak height and time‐to‐peak (Figure S14A‐C). Conversely, decreasing λ Exo slowed decay and prolonged pulses, extending FWHM from 13 to 65 min, again accompanied by shifts in peak metrics (Figure S15B,C). Notably, the EDRN reaction remained reliable under modest temperature fluctuations (Figure S16A‐F). EDRN can sense target over 5 nM (Figure S17A,B). To simulate EDRN kinetics, we derived effective rate constants from the experimental data and constructed a reaction framework that reproduced the key dynamic features of the system. In Figure S10B, simulations across different inputs closely matched the experimental trajectories, and in Figure S15A, simulations with varying λ Exo concentrations reproduced the corresponding experimental trends. This model is a lumped, system‐level approximation based on effective rate constants and does not explicitly capture enzyme activity decay, enzyme‐substrate dissociation, byproduct‐mediated inhibition, or off‐pathway reactions, which may contribute to discrepancies between simulations and experiments. It is therefore intended to describe system behavior and predict trends within the validated operating regime, rather than provide a complete mechanistic description of all enzymatic microstates. These results show a two‐step, faithful mapping in which stimulus identity is first translated into concentration states by the converter and then rendered as precise temporal signatures by λ Exo.

Collectively, these findings establish a robust and versatile strategy in which a Bst‐based concentration converter and a λ Exo‐driven temporal decoder operate sequentially to transform molecular identity into programmable time‐domain signals. By defining concentration as the central intermediate and Us as the standardized information carrier, this system supports all three fundamental encoding modes—same input producing different times, different inputs producing distinct times, and different inputs converging on the same time—thereby providing a generalizable foundation for temporal programming in synthetic DNA circuits.

### 2.3 Expanding Temporal Duration by Double‐layered Concentration Convertor

**FIGURE 4:**
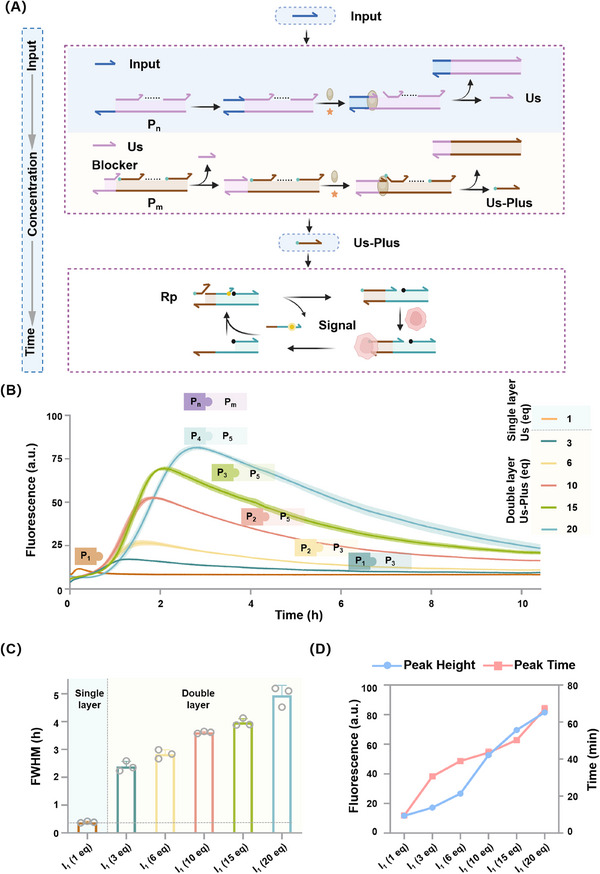
Multilayer concentration converter for the extension of temporal signals. (A) Schematic of the double‐layer concentration converter. The product of the first polymerase‐mediated extension‐displacement reaction serves as the input for a second converter, amplifying Us production. (B) Fluorescence curves of single‐layer and double‐layer converters. The sequential conversion markedly prolongs signal duration and shifts pulse envelopes to later timepoints. (C) FWHM extracted by panel B. The double‐layered converter markedly broadened the pulse duration. 1 eq belongs to single layer. (D) Analysis of peak value and time‐to‐peak. The increases in peak height and delayed peak timing relative to the single‐layer design. All experiments were performed with 50 nM I_1_, 50 nM P_n_, 200 nM P_m_, 1000 nM Rp, 160 U/mL Bst and 2.77 U/mL λ Exo in 1× Lambda reaction buffer at 25°C. Data are presented as mean ± SD (n = 3 independent experiments).

The dynamic range of temporal encoding with a single‐layer concentration converter is constrained by the finite amount of Us generated in a single polymerase extension‐displacement cycle. In addition, the maximum synthesis length of ssDNA (typically 150 nt with current oligonucleotide synthesis technologies) further restricts the yield of Us. To overcome these limitations, we designed a double‐layer concentration converter. In this configuration, the P_n_ first recognizes the input and, under the action of Bst, releases n number of Us. Us then serves as a trigger for the P_m_ module, where it initiates a secondary Bst‐mediated extension‐displacement reaction to release m number of extended products, Us‐Plus. It is noteworthy that a blocking strand (Blocker) within P_m_ prevents Us‐Plus from premature λ Exo digestion, ensuring it can act effectively on downstream modules (Figure 4A). Experiments show that the Blocker effectively suppresses leakage in the double‐layer concentration converter (Figure S18), and Bst polymerase enables efficient release of Us‐Plus (Figure S19A‐D). The accumulated Us‐Plus subsequently reacts with the reporter to generate pulse signals. The double‐layered concentration converter enables iterative amplification of the input, producing m × n copies of Us‐Plus, which in turn extend the duration of downstream pulses decoded by λ Exo. Kinetic measurements demonstrated that the double‐layer converter produced rise‐fall trajectories of significantly longer duration compared with the single‐layer design (Figure 4B). With the same input applied, pulse envelopes were consistently shifted to later timepoints, confirming that sequential processing provides an additional tier of temporal control. Analyses further revealed systematic enhancements in output features: the FWHM results indicate that the double‐layered concentration converter further expands the temporal window from 20 min to 5 h, achieving a shift from the minute to the hour timescale (Figure 4C). Both peak intensity and time‐to‐peak increased proportionally with the concentration of Us‐Plus (Figure 4D). Direct comparisons showed that pulse lifetimes were extended by more than 15‐fold relative to the single‐layer system, while waveform fidelity was preserved. By integrating these layers of control, DNA circuits can achieve scalable, distortion‐free modulation of molecular lifetimes across a substantially broadened temporal spectrum.

### 2.4 Multiplexing Detection of Bacterial Genes in Clinical Samples by Stimuli‐Dependent Temporal Molecular Pulse

Multiplexed detection of bacterial virulence and drug resistance gene markers is essential for guiding treatment and controlling infection spread. Clinical data demonstrate that genes encoding resistance determinants and virulence factors are tightly linked to patient outcomes [36, 37, 38, 39]. For example, *mecA* defines methicillin‐resistant *Staphylococcus aureus* (MRSA), which accounts for ∼20–40% of *S. aureus* isolates worldwide and is associated with mortality rates of 20–50% in bloodstream infections [40, 41]. Similarly, *vanA*/*vanB* confer vancomycin resistance in *Enterococcus faecium*/*faecalis*, with treatment failure rates exceeding 40% and outbreaks contributing to hospital mortality burdens [42, 43]. Among virulence genes, *pvl* is found in ∼5% of *S. aureus* isolates but is strongly enriched in community‐associated MRSA and linked to mortality rates >30% in necrotizing pneumonia cases [44, 45]. Genes such as *sek2*, *seq2*, and *arcA* are prevalent in invasive clinical isolates, with carriage rates of 20–35% in multidrug‐resistant strains, and contribute to immune evasion and tissue colonization [46, 47]. However, existing probe‐based methods face significant challenges: multiplexing is typically limited by spectral overlap, probe design complexity, and crosstalk between channels, making it difficult to detect multiple clinically important genes within a single tube [48].

**FIGURE 5:**
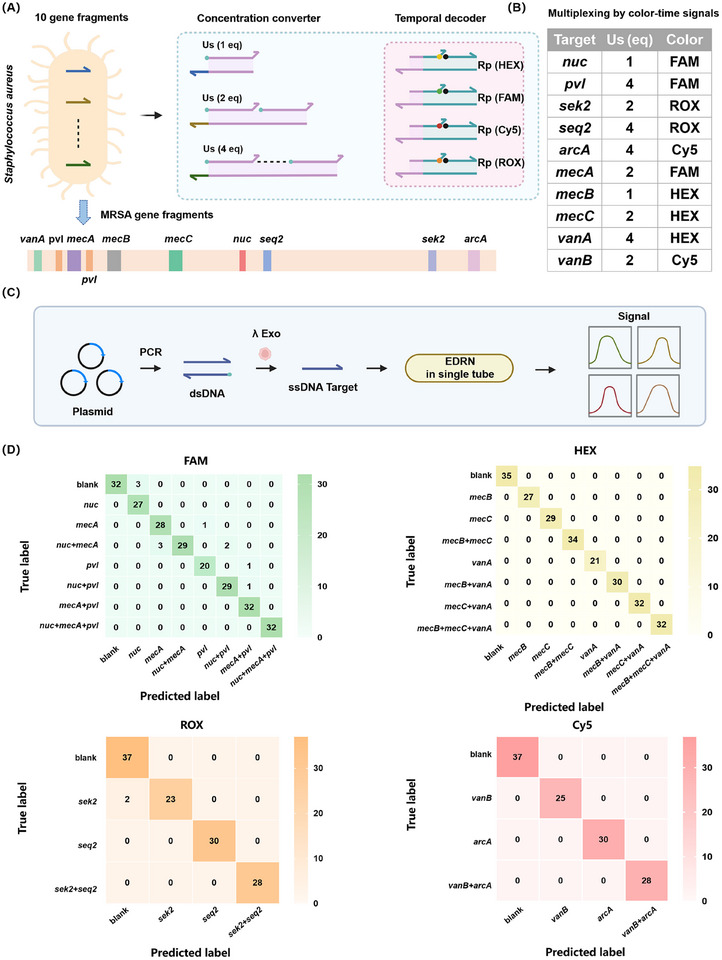
EDRN for multiplex bacterial nucleic acid detection. (A) Schematic illustration of the multiplex detection system. Multiplexed bacterial nucleic acid detection through concentration‐based temporal codes and four spectrally distinct reporters. (B) Encoding strategy for ten bacterial targets. A set of ten targets was encoded into four fluorescence channels with varying concentrations of Us. 20 nM Us was defined as 1 eq. (C) Procedure for target gene carried plasmid detection. Plasmid validation confirming reproducible fluorescence trajectories and reliable temporal encoding for multiplexed detection. (D) Confusion matrices of test set presented by fluorescence channels. All experiments were conducted with 20–140 nM Us, 140 nM Rp, 160 U/mL Bst and 8.33 U/mL λ Exo in 1× Lambda reaction buffer at 25°C.

To address these challenges, we applied EDRN to establish a multiplex bacterial nucleic acid detection system. Based on the reaction principle of EDRN, we implemented a combinatorial encoding strategy that uses three temporal windows across four fluorescence color channels, enabling discrimination of 12 targets in a single tube (Figure 5A; Figure S20). Each gene target was assigned a specific Us concentration and mapped to one of the reporters in four colors (Figure 5B). This approach transforms molecular identity into a decodable kinetic signal, generating a unique time‐color signature. Because EDRN encodes targets in kinetic signatures, the time‐to‐result can be shortened by increasing Bst polymerase and λ Exo to accelerate pulse rise and decay, and by optimizing the Rp toehold to speed strand displacement. This preserves the multiplexing scheme while compressing the temporal windows. The signals of the synthetic strands of ten critical gene fragments of MRSA and representative combinations are shown in Figure S21. The selection criteria for the ten gene sequences are described in the Supporting Notes. To ensure measurement accuracy, we confirmed the absence of crosstalk of fluorescence channel by using the concentration converter (Figure S22A,B). To further evaluate sequence specificity, we examined ten gene sequences containing mismatches. The concentration converter functions as the recognition module. The results show that 1 mismatch renders discriminative signal against full match, while 2 and more than 2 mismatches completely inhibits polymerase reaction (Figure S23). If developing EDRN for detecting single mutation in the future, the framework can be adapted by (i) leveraging feature‐based machine‐learning analysis of temporal trajectories to enhance separation of subtle kinetic differences, (ii) introducing blocker strands/clamps to suppress extension from single‐mismatched complexes in the concentration converter, (iii) selecting polymerases with higher mismatch discrimination (or engineered variants) and optimizing reaction stringency. To simulate clinical samples, plasmids carrying target gene fragments were amplified by PCR, converted to ssDNA via λ Exo digestion, and finally analyzed using EDRN (Figure 5C). The resulting fluorescence curves confirmed detection of the genes and representative combinations (Figure S24). Given the richness of pulse features (e.g., peak height, FWHM), we further applied machine learning classifiers to identify targets with high accuracy. Machine learning classification of experimental datasets yielded accuracies above 95%, with confusion matrices confirming robust recognition of multiplexed bacterial nucleic acids (Figure 5D; Figure S25). In the confusion matrix, rows denote true labels and columns denote predicted labels; unequal row totals reflect class imbalance in the test set, and row–column mismatches indicate misclassifications (false positives/negatives).

**FIGURE 6:**
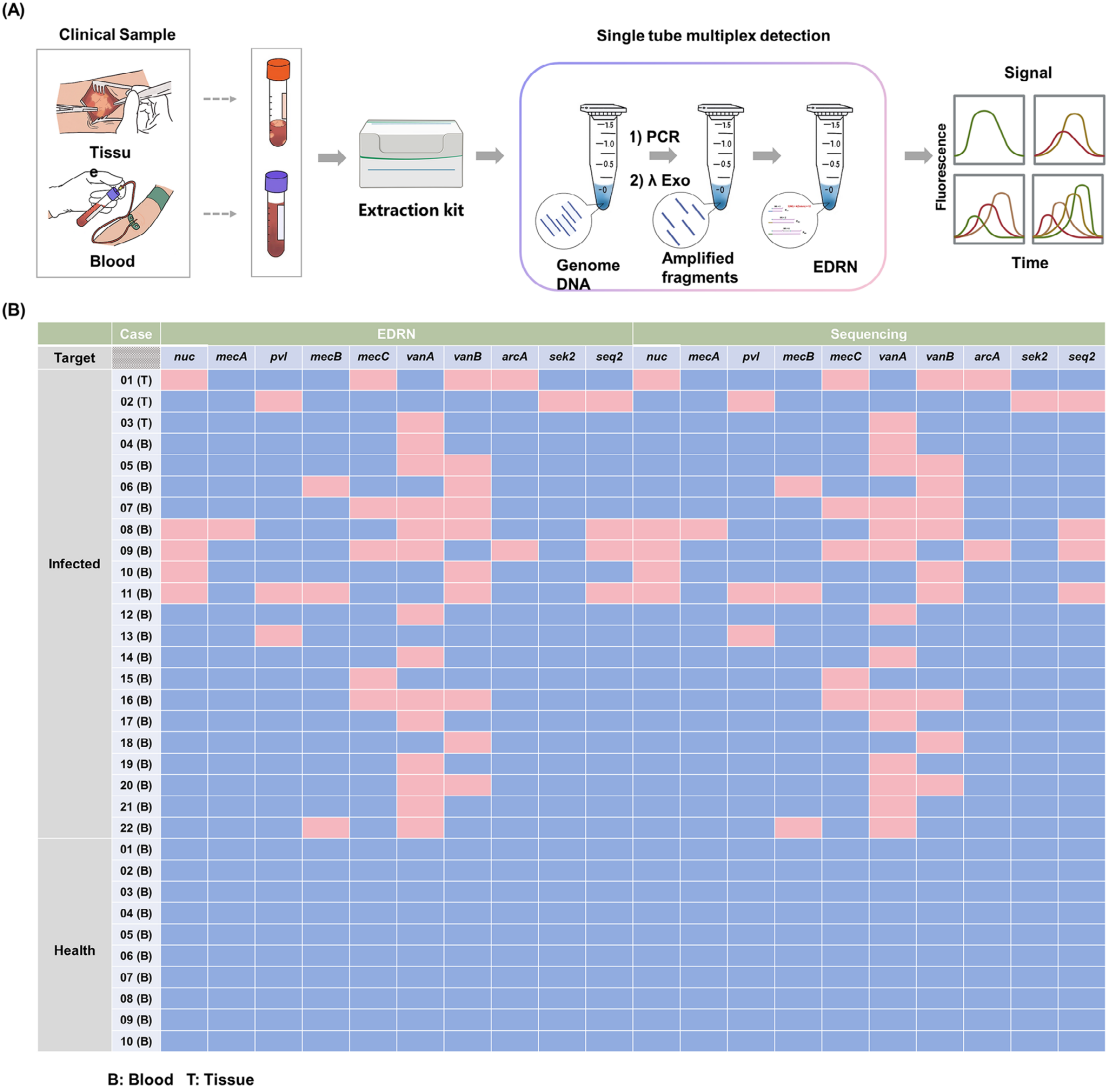
MRSA gene detection of clinical samples by EDRN. (A) Schematic workflow of the clinical detection. Blood and tissue samples were collected from infected patients and healthy donors, followed by genomic DNA extraction. Target gene regions were amplified by PCR and processed with λ Exo to yield single‐stranded DNA, which was then analyzed using EDRN to generate fluorescence curves. (B) Comparison of multiplex detection results (left matrix) and sequencing validation (right matrix). Ten target genes were analyzed across 32 clinical samples, including 22 infection‐positive cases and 10 healthy controls. Each row represents a patient case, and each column represents a gene target of MRSA. Red indicates positive detection. The high concordance between multiplex detection and sequencing validates the system's diagnostic accuracy and robustness in real clinical samples. All experiments were conducted with 20–140 nM Us, 140 nM Rp, 160 U/mL Bst and 8.33 U/mL λ Exo in 1× Lambda reaction buffer at 25°C.

We next assessed clinical performance using 32 patient‐derived samples (22 infection‐positive, 10 healthy controls). Blood and tissue specimens were processed through a standardized workflow encompassing nucleic acid extraction, amplification, and fluorescence kinetic detection (Figure 6A). Figure S26 demonstrates that, within the tested range, variation in the concentration of extracts from confirmed positive clinical samples, followed by PCR amplification, has no measurable effect on the EDRN readout. After confirming that the template input was sufficient, we performed the sample analysis. Sequencing validation of the ten target genes revealed a high level of consistency, with a detection accuracy of 100%: infection‐positive cases exhibited distinct gene‐specific patterns, while all healthy controls remained negative (Figure 6B). Figures S27 and S28 show the fluorescence kinetic traces of the clinical samples. Gene‐level analysis of positive samples revealed distinct patterns in virulence and resistance targets (Figure S29A,B). Among the ten target genes, *vanA* and *vanB* exhibited relatively high prevalence. Overall, high‐resistance genes accounted for a larger proportion. These findings highlight the clinical and epidemiological significance of these molecular markers. Virulence and resistance gene profiling is critical for clinical decision‐making, yet current probe‐based assays remain poorly suited for high‐level multiplexing in a single tube. Our EDRN approach, through time‐color co‐encoding, overcomes these limitations and enables simultaneous, high‐fidelity detection of multiple clinically significant genes in single test.

In the current multiplex configuration, EDRN is designed primarily for qualitative identity decoding via discretized time–color signatures and is not intended to simultaneously provide robust quantification across targets, because the temporal codes are chosen to maximize separability rather than preserve a linear abundance readout. If semi‐quantitative information is desired, a practical two‐step workflow is to first use multiplex EDRN to identify which targets are present, and then infer relative abundance upstream through calibrated amplification conditions (e.g., controlling PCR cycle number) before running EDRN under standardized decoding conditions.

## 3 Conclusions

In conclusion, we establish an enzymatic DNA reaction network (EDRN) that enables temporal encoding to function as an explicit and programmable information axis in cell‐free nucleic acid systems. By decoupling stimulus recognition from temporal programming through a polymerase‐based concentration converter and an exonuclease‐driven temporal decoder, EDRN allows molecular identity to be translated into well‐defined temporal signatures with predictable control over pulse amplitude and lifetime. This modular design avoids reliance on delicate DNA structure–based kinetic tuning and supports flexible reassignment, divergence, and convergence of temporal behaviors, positioning time as a rationally engineerable output rather than a fixed byproduct of molecular recognition.

A key practical feature of EDRN is its ability to expand multiplexing capacity beyond the limits of fluorescence‐based multiplexed assays [10]. EDRN is not intended to replace amplification, but to serve as a high‐density readout layer that increases multiplex capacity by combining dye channels with programmable temporal windows, thereby reducing the need to split broad panels across multiple parallel reactions. This addresses a persistent barrier to translating single‐analyte cell‐free biosensors into panel‐level tests [49]. For future point‐of‐care translation, the upstream workflow can be streamlined by coupling EDRN with rapid lysis/extraction and/or isothermal amplification to reduce handling steps and instrumentation [50]. By combining temporal signatures with standard fluorescence color channels, EDRN substantially increases information density per assay. This addresses a persistent barrier to translating single‐analyte cell‐free biosensors into panel‐level tests, where multiplexing is constrained not only by finite fluorescence channels and spectral cross‐talk but also by the increasing interaction complexity of one‐pot mixtures [51], which can introduce off‐target reactions and target‐dependent response bias. In our implementation, four commonly used fluorophores paired with three programmable temporal windows enable discrimination of up to twelve targets in a single tube, reducing the need for multiple parallel reactions and thereby lowering reagent consumption, sample input, and workflow complexity. Accordingly, EDRN should be viewed as a scalable readout/encoding layer that complements‐rather than replaces‐front‐end amplification strategies. In this sense, EDRN complements existing amplification strategies by providing a scalable and information‐rich readout framework.

We also recognize the current limitations of the system and outline directions for future development. The present implementation relies on a multi‐step workflow and a largely linear, externally parameterized architecture, which favors programmability and interpretability but offers limited intrinsic buffering under non‐ideal conditions. In particular, unlike many cellular networks [52], the current decoder does not yet incorporate nonlinear self‐correcting feedback, which may affect robustness under perturbations such as temperature variation, enzyme‐activity drift, or complex sample matrices. Moreover, while this study focuses on conserved regions for robust gene‐level detection, SNP‐oriented applications could be enabled by exploiting the mismatch sensitivity of polymerase extension and by adding auxiliary specificity elements (e.g., blocker strands or more mismatch‐discriminating polymerases) [53]. Combined with feature‐based analysis of temporal trajectories, such adaptations may ultimately support multiplex SNP calling within the same time–color encoding framework. Future efforts will focus on integrating EDRN with simplified upstream handling and isothermal amplification to improve point‐of‐care compatibility, reducing instrumentation and operational complexity, as well as exploring feedback‐enabled or self‐regulating designs to enhance robustness. In addition, temporal window selection can be tailored to balance multiplexing capacity with assay turnaround time, enabling adaptation to diverse application scenarios. Assay turnaround time can be further reduced by accelerating pulse rise and decay through enzyme‐level tuning (e.g., Bst polymerase and λ Exo) and by optimizing strand‐displacement kinetics (e.g., Rp toehold design), while preserving the time–color multiplexing scheme. Finally, coupling EDRN with feature‐based analysis (e.g., machine‐learning classification of temporal trajectories) provides a route toward automated calling for high‐order panels, which will be important for practical deployment. Collectively, these advances will further extend the utility of temporal encoding as a general strategy for scalable, expressive, and practical molecular diagnostics and synthetic biochemical circuits.

## 4 Experimental Section

### 4.1 Reagents and Enzymes

All DNA oligonucleotides were synthesized by Sangon Biotech (Shanghai, China) and purified via PAGE, ULTRAPAGE, or HPLC. Enzymes, reaction buffers, and dNTPs (2.5 mM) were obtained from New England Biolabs (NEB). Plasmids and microbial DNA isolation kits were supplied by QIAGEN. All experiments were conducted using DNase‐/RNase‐free water (Tiangen Biotech, Beijing, China).

### 4.2 EDRN Preparation and Operation

DNA oligonucleotides were diluted to 100 µM in DNase/RNase‐free deionized water for use. All DNA structures were prepared separately. For example, corresponding oligonucleotides were mixed in 1×Lambda reaction buffer (67 mM glycine‐KOH, 2.5 mM MgCl_2_, 50 µg/mL BSA, pH 9.4), and annealed from 90 to 4°C at a rate of 1°C min^−1^. Within the concentration converter and temporal decoder, P_n_ and Rp were first subjected to independent annealing processes, after which the required strands were mixed together in the reaction system.

### 4.3 Characterization of the DNA Reactions by Polyacrylamide Gel Electrophoresis

The products of DNA‐based reactions were characterized by 20 % native PAGE, which was run in TBE buffer (89 mM Tris‐Borate, 2 mM EDTA, pH 8.0) at 4°C at a voltage of 120 V for 3 h. After 20 min of staining in SYBR GOLD (Invitrogen) dissolved in 0.5×TBE buffer, the gel was photographed using a gel imaging system (Tanon‐2500BR).

### 4.4 Real‐Time Fluorescence Assays

The probes were prepared by mixing the corresponding DNA oligonucleotides in 1*×*Lambda reaction buffer in a 50 µL system. After the addition of the input, the fluorescence was recorded immediately by a real‐time fluorescence quantitative PCR instrument (Rotor‐Gene Q, Qiagen, Germany) at 25°C with an interval of 5 s.

### 4.5 PCR and Genomic DNA Extraction From Clinical Samples

PCR was performed in 50 µL system including 1 µL extracted DNA, 10 µL dNTP (2.5 µM), 2.5 µL forward primer (10 µM), 2.5 µL reverse primer (10 µM), 5 µL PCR buffer, and 1 µL Taq DNA Polymerase with Standard Taq Buffer. Thermal cycling consisted of 98°C for 30 s, followed by corresponding cycle (e.g., *nuc* 20) of 98°C for 10 s, corresponding temperature for 30 s (e.g., *nuc* 52°C), and 72°C for 30 s, finally 72°C for 2 min and cooling to room temperature. To generate single‐stranded DNA (ssDNA) of the amplification product, the PCR product was digested with λ Exo (0.4 U/µL) for 1 h, followed by heating at 90°C for 10 min to inactivate the enzyme. In accordance with biosafety requirements, nucleic acids from bacterial infection samples were extracted in our BSL‐2 laboratory. This study was approved by the Ethics Committee of Tianjin Hospital of Tianjin University (ethics number: 2024–149). Total DNA was isolated from 200 µL of blood or 30 mg of tissue using the QIAamp DNA Blood or Tissue Kit (QIAGEN) according to the manufacturer's instructions. The extracted DNA is stored at −80°C for later use.

### 4.6 Machine Learning

Raw fluorescence trajectories were first converted into estimated concentrations of free fluorescent strands using the equation (F_t_−F_min_)/(F_max_−F_min_)×[Signal], where F_t_ is the fluorescence intensity at each time point, F_max_ and F_min_ are the fluorescence intensities of the free and hybridized fluorescent strands, respectively, and [Signal] is the concentration of the fluorophore‐labeled signal strand. Pulse‐derived kinetic features, including peak height and full width at half maximum (FWHM), were then extracted from the processed trajectories and organized into pulse‐descriptor vectors. These features were assembled into a sample‐by‐feature matrix and standardized by Z‐score normalization (zero mean, unit variance). The scaler was fitted on the training set and subsequently applied to the test set to ensure consistent feature transformation. Although principal component analysis (PCA) was considered as an optional dimensionality‐reduction step, all reported analyses were performed using the full standardized feature set to retain the kinetic information encoded in the pulse‐like signals. Classification was conducted using a Random Forest model implemented in scikit‐learn (Python) with 100 decision trees (n_estimators = 100). Class labels were assigned by majority voting across trees. Model performance was evaluated using a reproducible hold‐out strategy, in which the dataset was randomly divided into training (70%) and test (30%) subsets with a fixed random state. The model was trained exclusively on the training set, and predictive performance on the held‐out test set was assessed using accuracy, weighted precision, weighted recall, and weighted F1‐score. Confusion matrices were used to visualize class‐specific errors and misclassification patterns. Because the held‐out test set was not perfectly class‐balanced, the row totals in the confusion matrix may differ across classes. This evaluation reflects internal generalization to the held‐out portion of the same dataset. Additional details are provided in the Supporting Notes. Source code is available at https://github.com/Su‐Laboratory/ML.git.

## Conflicts of Interest

The authors declare no conflicts of interest.

## Supporting information




**Supporting File**: advs75343‐sup‐0001‐SuppMat.pdf.

## Data Availability

The data that support the findings of this study are available from the corresponding author upon reasonable request.
