# Supplementary material for: Enzymatic DNA Reaction Networks for Orchestrating Stimuli‐Dependent Temporal Molecular Pulse
Source: Adv Sci (Weinh). 2026 Apr 20;13(38):e20984. doi: 10.1002/advs.202520984 (PMC13335520; doi:10.1002/advs.202520984)
Supplement: Supplementary file 1 — Supporting File: advs75343‐sup‐0001‐SuppMat.pdf. [file ADVS-13-e20984-s001.pdf]

Supporting Information

Enzymatic DNA Reaction Networks for Orchestrating Stimuli-Dependent Temporal Molecular Pulse

Jiayu Yang, Yali Chang, Zibin Chu, Linghao Zhang, Tengfang Zhang, Dai Erhei, Xin Su, and Zhe Yin**

J. Yang, Y. Chang, Z. Chu, L. Zhang, T. Zhang, Prof. X. Su.
State Key Laboratory of Organic-Inorganic Composites, Beijing Key Laboratory of Bioprocess, Beijing Advanced Innovation Center for Soft Matter Science and Engineering, College of Life Science and Technology, Beijing University of Chemical Technology, Beijing, China.
E-mail: xinsu@mail.buct.edu.cn
J. Yang and Y. Chang contributed equally.

Prof. Z. Yin.
State Key Laboratory of Pathogen and Biosecurity, Academy of Military Medical Sciences, Beijing, China.
E-mail: jerry9yin@163.com

Prof. D. E.
Hebei Key Laboratory of immune Mechanism of Major Infectious Diseases and New Technology of Diagnosis and Treatment, The Fifth Hospital of Shijiazhuang, Hebei Medical University, Shijiazhuang, China.

* Corresponding author

This PDF file includes:

Supporting Table: Table S1
Supporting notes: 1-3
Supporting Figures: Figure S1-S29

Supporting Tables

Table S1. Sequence of the oligonucleotides in this work.

Sequence of the concentration converter		
**Same input-Different concentrations**		
Name	Sequence (5' -3')	Purification
I_1	GGACAAATTGCAAATCCAGAAAGTTT	HPLC
SD_1	C*G*T*C*GCCAGGCACCGGAGCGAAACTT TCTGGATTTGCAATTTGTCCT*T*T*T*T	HPLC
SD_2	C*G*T*C*GCCAGGCACCGGAGCGCGTCGC CAGGCACCGGAGCGAAACTTTCTGGATTT GCAATTTGTCCT*T*T*T*T	HPLC
SD_3	C*G*T*C*GCCAGGCACCGGAGCGCGTCGC CAGGCACCGGAGCGCGTCGCCAGGCACC GGAGCGAAACTTTCTGGATTTGCAATTTGT CCT*T*T*T*T	HPLC
SD_4	C*G*T*C*GCCAGGCACCGGAGCGCGTCGC CAGGCACCGGAGCGCGTCGCCAGGCACC GGAGCGCGTCGCCAGGCACCGGAGCGAA ACTTTCTGGATTTGCAATTTGTCCT*T*T*T* T	HPLC
Us	P-CGCTCCGGTGCCTGGCGACGTTTTT	HPLC
**Different inputs-Different concentrations**		
I_1	GGACAAATTGCAAATCCAGAAAGTTT	HPLC
I_2	AAAGGCTGAAAACGGAAAAGATCTTCA	HPLC
I_3	CATGGGGAACGAGGATGATTTGATTGTC	HPLC
I_4	AACGACAATTGCTATTCAGCTGTACTCTCG	HPLC
DD_1	C*G*T*C*GCCAGGCACCGGAGCGAAACTT TCTGGATTTGCAATTTGTCCT*T*T*T*T	HPLC
DD_2	C*G*T*C*GCCAGGCACCGGAGCGCGTCGC CAGGCACCGGAGCGTGAAGATCTTTTCCG TTTTCAGCCTTTT*T*T*T*T	HPLC
DD_3	C*G*T*C*GCCAGGCACCGGAGCGCGTCGC CAGGCACCGGAGCGCGTCGCCAGGCACC	HPLC

DD_4	GGAGCGGACAATCAAATCATCCTCGTTCCC<br>CATGT*T*T*T*T<br>C*G*T*C*GCCAGGCACCGGAGCGCGTCGC<br>CAGGCACCGGAGCGCGTCGCCAGGCACC<br>GGAGCGCGTCGCCAGGCACCGGAGCGCG<br>AGAGTACAGCTGAATAGCAATTGTCGTTT*<br>T*T*T*T	HPLC
Us	P-CGCTCCGGTGCCTGGCGACGTTTTT	HPLC
**Different inputs-Same concentration**		
I_1	GGACAAATTGCAAATCCAGAAAGTTT	HPLC
I_2	AAAGGCTGAAAACGGAAAAGATCTTCA	HPLC
I_3	CATGGGGAACGAGGATGATTTGATTGTC	HPLC
I_4	AACGACAATTGCTATTCAGCTGTACTCTCG	HPLC
DS_1	C*G*T*C*GCCAGGCACCGGAGCGAAACTT<br>TCTGGATTTGCAATTTGTCCT*T*T*T*T	HPLC
DS_2	C*G*T*C*GCCAGGCACCGGAGCGTGAAGA<br>TCTTTTCCGTTTTCAGCCTTTT*T*T*T*T	HPLC
DS_3	C*G*T*C*GCCAGGCACCGGAGCGGACAAT<br>CAAATCATCCTCGTTCCCCATGT*T*T*T*T	HPLC
DS_4	C*G*T*C*GCCAGGCACCGGAGCGCGAGA<br>GTACAGCTGAATAGCAATTGTCGTTT*T*T*<br>T*T	HPLC
Us	P-CGCTCCGGTGCCTGGCGACGTTTTT	HPLC

Note: Probe $P_{1\text{-}SD}$ is composed of the SD_1 and Us; Probe $P_{1\text{-}DD}$ is composed of the DD_1 and Us; Probe $P_{1\text{-}DS}$ is composed of the DS_1 and Us.

**Sequence of the temporal decoder**		
Name	Sequence (5' -3')	Purification
Signal	TGGCGACGTTTTTACCTCT(HEX)TTTTT	HPLC
BHQ	(BHQ1)GCTAGACGCAGATGTACTGTCTGTA	HPLC
RecH	T*A*C*A*GACAGTACATCTGCGTCTAGCA<br>GAGGTAAAAAACGTCGCCAGGCACCGG*A<br>*G*C*G	HPLC

Note: Rp is composed of the Signal, BHQ and RecH.

Sequence of the double-layered concentration convertor		
Name	Sequence (5' -3')	Purification
Us'	CGCTCCGGTGCCTGGCGACGTTTTT	HPLC
C-Us	P-CGTTGCGAGTGATGGACTGCTTTTT	HPLC
Blocker	GTGCCTGGCGACGTTTTTTTTTT	HPLC
C-P_3	G*C*A*G*TCCATCACTCGCAACGGCAGTC CATCACTCGCAACGGCAGTCCATCACTCG CAACGAAAAACGTCGCCAGGCACCGG*A* G*C*G	HPLC
C-P_5	G*C*A*G*TCCATCACTCGCAACGGCAGTC CATCACTCGCAACGGCAGTCCATCACTCG CAACGGCAGTCCATCACTCGCAACGGCAG TCCATCACTCGCAACGAAAAACGTCGCCA GGCACCGG*A*G*C*G	HPLC
C-HEX	TGGACTGCTTTTTACCTCT(HEX)TTTTT	HPLC
C-BHQ	(BHQ1)GCTAGACGCAGATGTACTGTCTGTA	HPLC
C-RecH	T*A*C*A*GACAGTACATCTGCGTCTAGCA GAGGTAAAAAGCAGTCCATCACTCGC*A *A*C*G	HPLC

Note: P_3 (P_m) is conmposed of the C-Us, Blocker and C-P_3.

Sequence of the multiple detection system		
Name	Sequence (5' -3')	Purification
Us-FAM	P-CGTTGCACACGCCCTGGCTTTTT	HPLC
F-1	G*C*C*A*GGGCGTGTGCAACGCTTCAGGG CCATATTTCTCTACACCT*T*T*T*T	HPLC
F-2	G*C*C*A*GGGCGTGTGCAACGGCCAGGGC GTGTGCAACGGACGTCATATGAAGGTGTG CTTACAAGTT*T*T*T*T	HPLC
F-4	G*C*C*A*GGGCGTGTGCAACGGCCAGGGC GTGTGCAACGGCCAGGGCGTGTGCAACGG	HPLC

	CCAGGGCGTGTGCAACGAGGTGTGATATG TTGAGCTCGATCAT*T*T*T*T	
F-shelf	T*A*C*A*GACAGTACATCTGCGTCTAGCA AAAAGCCAGGGCGTGTGC*A*A*C*G	HPLC
F	CACGCCCTGGCTTTTT(FAM)TTTTT	HPLC
Us-HEX	P-CGCTCCGGTGCCTGGCGACGTTTTT	HPLC
H-1	C*G*T*C*GCCAGGCACCGGAGCGAAACTT TCTGGATTTGCAATTTGTCCT*T*T*T*T	HPLC
H-2	C*G*T*C*GCCAGGCACCGGAGCGCGTCGC CAGGCACCGGAGCGTGAAGATCTTTTCCG TTTTCAGCCTTTT*T*T*T*T	HPLC
H-4	C*G*T*C*GCCAGGCACCGGAGCGCGTCGC CAGGCACCGGAGCGCGTCGCCAGGCACC GGAGCGCGTCGCCAGGCACCGGAGCGGC GCCCTTGCGTTGTACCCGCGAGAGTACAG CTGAATAGCAATTGTCGTTT*T*T*T*T	HPLC
H-shelf	T*A*C*A*GACAGTACATCTGCGTCTAGCA GAGGTAAAAAACGTCGCCAGGCACCGG*A *G*C*G	HPLC
H	TGGCGACGTTTTTTACCTCT(HEX)TTTTT	HPLC
Us-Cy5	P-CGTTGGACGCGTTCACTCGCTTTTT	HPLC
C-2	G*C*G*A*GTGAACGCGTCCAACGGCGAGT GAACGCGTCCAACGGACAATCAAATCATC CTCGTTCCCCATGT*T*T*T*T	HPLC
C-4	G*C*G*A*GTGAACGCGTCCAACGGCGAGT GAACGCGTCCAACGGCGAGTGAACGCGTC CAACGGCGAGTGAACGCGTCCAACGGATT CTCTACGTCGTGCTCTCCAATT*T*T*T*T	HPLC
C-shelf	T*A*C*A*GACAGTACATCTGCGTCTAGCA GATAAAAAAGCGAGTGAACGCGTCC*A*A *C*G	HPLC
C	TCACTCGCTTTTTTATCT(Cy5)TTTTT	HPLC
Us-ROX	P-CGTTGCGAGTGATGGACTGCTTTTT	HPLC
R-2	G*C*A*G*TCCATCACTCGCAACGGCAGTC	HPLC

R-4	CATCACTCGCAACGATATTCGTTAGTAGCT GTGACTCCAT*T*T*T*T G*C*A*G*TCCATCACTCGCAACGGCAGTC CATCACTCGCAACGGCAGTCCATCACTCG CAACGGCAGTCCATCACTCGCAACGTTCC AGAACTAACTCCTTGAAGCGTT*T*T*T*T	HPLC
R-shelf	T*A*C*A*GACAGTACATCTGCGTCTAGCA GAGGTAAAAAAGCAGTCCATCACTCGC*A *A*C*G	HPLC
R	TGGACTGCTTTTTTACCTCT(ROX)TTTTT	HPLC
Q	(BHQ1)GCTAGACGCAGATGTACTGTCTGTA	HPLC

Note: P_{M1} (FAM) is conmposed of the Us-FAM and F-1; Probe RpF is conmposed of the F-shelf, F and Q.

**Sequence of targets**		
nuc	GGTGTAGAGAAATATGGCCCTGAAG	HPLC
mecA	ACTTGTAAGCACACCTTCATATGACGTC	HPLC
mecB	GGACAAATTGCAAATCCAGAAAGTTT	HPLC
mecC	AAAGGCTGAAAACGGAAAAGATCTTCA	HPLC
vanA	AACGACAATTGCTATTCAGCTGTACTCTCG	HPLC
vanB	CATGGGGAACGAGGATGATTTGATTGTC	HPLC
pvl	TGATGCAGCTCAACATATCACACCT	HPLC
sek2	TGGAGTCACAGCTACTAACGAATAT	HPLC
seq2	ACGCTTCAAGGAGTTAGTTCTGGAA	HPLC
arcA	ATTGGAGAGCACGACGTAGAGAATC	HPLC

**Sequence of PCR**		
nuc-Fp	GCAAAGGTCAACCAATGACATTCAGACTA	HPLC
nuc-Rp	P-CTTCAGGGCCATATTTCTCTACACCT	HPLC
nuc	CTTCAGGGCCATATTTCTCTACACCTTTTTT AGGATGCTTTGTTTCAGGTGTATCAACTAA TAATAGTCTGAATGTCATTGGTTGACCTTT	HPLC

	G	
mecA-Fp	CGGATTATGGCTCAGGTACTGCTAT	HPLC
mecA-Rp	P-GACGTCATATGAAGGTGTGCTTACAAG	HPLC
mecA	GACGTCATATGAAGGTGTGCTTACAAGTGC TAATAATTCACCTGTTTGAGGGTGGATAGC AGTACCTGAGCCATAATC	HPLC
pvl-Fp	GGGAAGTGAACTGGAAAACTCATGAAAT	HPLC
pvl-Rp	P-AGGTGTGATATGTTGAGCTGCATC	HPLC
pvl	AGGTGTGATATGTTGAGCTGCATCAAGTGT ATTGGATAGCAAAAGCAATGCAATTGATGT AACTTCTCTAGATTTGACTATTTTTTCATA TCAATTATGTCCTTTCACTTTAATTTCATGA GTTTTCCAGTTCACTT	HPLC
mecB-Fp	GGGCATGAAAGCTTTAGACATTGG	HPLC
mecB-Rp	P-AAACTTTCTGGATTTGCAATTTGTCCT	HPLC
mecB	AAACTTTCTGGATTTGCAATTTGTCCTTTTT GAAAATAATAATCAGAAGGAATATTTCTCC CAATGTCTAAAGCTTTCATGCCC	HPLC
mecC-Fp	GCAAAACACTGATGGTTTTAAGGTATCC	HPLC
mecC-Rp	P-TGAAGATCTTTTCCGTTTTCAGCCT	HPLC
mecC	TGAAGATCTTTTCCGTTTTCAGCCTTTTTCT CCAATAATGTGTCTAAAGGTTTATTGTCATA AGTATTTGCAATGGATACCTTAAAACCATC AGTGTTTTGC	HPLC
vanA-Fp	ACGAGCCGTTATACATTGGAATTACG	HPLC
vanA-Rp	P-CGAGAGTACAGCTGAATAGCAATTG	HPLC
vanA	CGAGAGTACAGCTGAATAGCAATTGTCGTT TTCCCATTCCGCGCAAGGTTTTTCGCACAT TTTCCATACACCAGATTTCGTAATTCCAATG TATAACGGCTCGT	HPLC
vanB-Fp	TTAACGCTGCGATAGAAGCGG	HPLC
vanB-Rp	P-GACAATCAAATCATCCTCGTTCCCCA	HPLC
vanB	GACAATCAAATCATCCTCGTTCCCCATGAC CGCACACCCGACCTCACAGCCCGAAATCG	HPLC

	CTTGCTCAATTAAGATTTTTCCATCATATTG TCCTGCCGCTTCTATCGCAGCGTTAA	
arcA-Fp	GCCCAACCTTTATTTTACAAGAGATCCC	HPLC
arcA-Rp	P-GATTCTCTACGTCGTGCTCTCCAAT	HPLC
arcA	GATTCTCTACGTCGTGCTCTCCAATACATTC TGTTAATTGTCATTCCTCTACCAATTGAAG CTTGGGGATCTCTTGTAAAATAAAGGTTGG GC	HPLC
sek2-Fp	GATGTTTTTGGTATTAGTTATAATGGCCAG	HPLC
sek2-Rp	P-AGGTGTGATATGTTGAGCTGCATC	HPLC
sek2	ATATTCGTTAGTAGCTGTGACTCCACCATAT ATGTATTTATTGTTACACTGGCCATTATAAC TAATACCAAAAACATC	HPLC
seq2-Fp	GGCACTGTTAGCTTGTTTTTCTTCACAT	HPLC
seq2-Rp	P-TTCCAGAACTAACTCCTTGAAGCGT	HPLC
seq2	TTCCAGAACTAACTCCTTGAAGCGTTTCTG GTTGATAATTAGTATAAAAGTTTCTAAGGTT GATTACCCCTACATCAGCATATGCTAGATTG TTTTTTATTAAAAATGTGAAGAAAAACAAG CTAACAGTG	HPLC

Note: *nuc*-Fp is used as the upstream primer; *nuc*-Rp is used as the reverse primer; *nuc* is used as the template.

**Sequence of perfect-match and mismatch variants**		
mecB-PM	GGACAAATTGCAAATCCAGAAAGTTT	HPLC
mecB-1nt-MM	GGACAAATTGCAAATCCAGAAAGTTC	HPLC
mecB-2nt-MM-1	GGACAAACTGCAAATCCAGGAAGTTT	HPLC
mecB-2nt-MM-2	GGACGAATTGCAAATCCAGAAAGCTT	HPLC
mecB-2nt-MM-3	GGACAAATTGTAAATCCGGAAAGTTT	HPLC
mecB-3nt-MM	GGACGAATTGCGAATCCAAAAAGTTT	HPLC
mecC-PM	AAAGGCTGAAAACGGAAAAGATCTTCA	HPLC
mecC-2nt-MM-1	AAAGGCTAAAAACGGAAAAAATCTTCA	HPLC
mecC-2nt-MM-2	AAAGGCTGAAGACGGAAAAGATCCTCA	HPLC
mecC-2nt-MM-3	AAAGACTGAAAACGGGAAAGATCTTCA	HPLC

vanA-PM	AACGACAATTGCTATTCAGCTGTACTCTCG	HPLC
vanA-2nt-MM-1	AACGACAACTGCTATTCAGCTATACTCTCG	HPLC
vanA-2nt-MM-2	AACGATAATTGCTATTCGGCTGTACTCTCG	HPLC
vanA-2nt-MM-3	AACGACAATTGCCATTCAGCTGTACTTTCG	HPLC
nuc-PM	GGTGTAGAGAAATATGGCCCTGAAG	HPLC
nuc-2nt-MM-1	GGTGTAGGGAAATATGGCCTTGAAG	HPLC
nuc-2nt-MM-2	GGTATAGAGAAATATGACCCTGAAG	HPLC
nuc-2nt-MM-3	GGTGTAGAGAGATATGGCCCCGAAG	HPLC
mecA-PM	ACTTGTAAGCACACCTTCATATGACGTC	HPLC
mecA-2nt-MM-1	ACTTGTAAGTACACCTTCATACGACGTC	HPLC
mecA-2nt-MM-2	ACTTGCAAGCACACCTTCATATGATGTC	HPLC
mecA-2nt-MM-3	ACTTGTAAGCATACCTTCATATAACGTC	HPLC
pvl-PM	TGATGCAGCTCAACATATCACACCT	HPLC
pvl-2nt-MM-1	TGATGCAACTCAACATATCGCACCT	HPLC
pvl-2nt-MM-2	TGATACAGCTCAACATATCACATCT	HPLC
pvl-2nt-MM-3	TGATGCAGCTTAACATATCATACCT	HPLC
vanB-PM	CATGGGGAACGAGGATGATTTGATTGTC	HPLC
vanB-2nt-MM-1	CATGGGGGACGAGGATGATTCGATTGTC	HPLC
vanB-2nt-MM-2	CATGAGGAACGAGGACGATTTGATTGTC	HPLC
vanB-2nt-MM-3	CATGGGGAACGGGGATGATTTGATCGTC	HPLC
arcA-PM	ATTGGAGAGCACGACGTAGAGAATC	HPLC
arcA-2nt-MM-1	ATTGGAAAGCACGACGTAGGGAATC	HPLC
arcA-2nt-MM-2	ATTAGAGAGCACGATGTAGAGAATC	HPLC
arcA-2nt-MM-3	ATTGGAGAGTACGACGTAGAGGATC	HPLC
sek2-PM	TGGAGTCACAGCTACTAACGAATAT	HPLC
sek-2nt-MM-1	TGGAATCACAGCTACTAACAAATAT	HPLC
sek-2nt-MM-2	TGGAGTCATAGCTACTAACGAGTAT	HPLC
sek-2nt-MM-3	TGAAGTCACAGCTATTAACGAATAT	HPLC
seq2-PM	ACGCTTCAAGGAGTTAGTTCTGGAA	HPLC
seq2-2nt-MM-1	ACGCTCCAAGGAGTTAGTTTTGGAA	HPLC
seq2-2nt-MM-2	ACGCTTCAAAGAGTTAGTTCTGAAA	HPLC
seq2-2nt-MM-3	ACACTTCAAGGAGCTAGTTCTGGAA	HPLC

Supporting Notes

1. Sequence selection criteria for the synthetic strands：

The synthetic target strands were selected using a set of design criteria intended to ensure efficient operation in the concentration-converter module while maintaining clinical relevance and analytical specificity. Target fragments were designed to be approximately 20-30 nucleotides in length, which matches the recognition/toehold span needed to reliably initiate strand displacement. Sequences were drawn from clinically relevant genes, including S. aureus species-specific markers, virulence-associated loci, and antibiotic-resistance determinants (with full target assignments provided in Table S1). For each gene, the chosen fragment was taken from a conserved region to support robust recognition across different strains and isolates. Candidate regions were also screened to minimize predicted secondary structure (e.g., hairpins or self-dimers) under assay conditions, thereby preserving accessibility and avoiding slowed strand-displacement kinetics. To reduce off-target triggering, sequence specificity was further evaluated using BLAST-based screening, excluding fragments with substantial similarity to non-target genes or organisms. Finally, sequences were checked for compatibility with the converter probe architecture, avoiding problematic repeats and extreme GC content that could destabilize intended hybridization and branch-migration behavior.

2. Reaction model and kinetics simulation of EDRN

Reaction equations:

$\mathrm{Input}+\mathrm{P_n} \xrightarrow{k_1} \mathrm{IP}$

$\mathrm{IP}+\mathrm{Bst} \xrightarrow{k_2} \mathrm{IP\text{-}Bst}$

$\mathrm{IP\text{-}Bst} \xrightarrow{k_3} \mathrm{W_{s1}}+\mathrm{Us}+\mathrm{Bst}$

$\mathrm{Us}+\mathrm{Rp} \xrightarrow{k_4} \mathrm{Signal}+\mathrm{Rp\text{-}Us}$

$\mathrm{Rp\text{-}Us}+\lambda\mathrm{Exo} \xrightarrow{k_5} \mathrm{Rp\text{-}Us\text{-}}\lambda$

$\mathrm{Rp\text{-}Us\text{-}}\lambda \xrightarrow{k_6} \mathrm{W_{s2}}+\mathrm{Rc}+\lambda\mathrm{Exo}$

$\mathrm{Rc}+\mathrm{Signal} \xrightarrow{k_7} \mathrm{Rp}$

Differential equations:

$d[\mathrm{Input}]/d[t] = -k_1 [\mathrm{Input}][\mathrm{P_n}]$

$d[\mathrm{P_n}]/d[t] = -k_1 [\mathrm{Input}][\mathrm{P_n}]$

$d[IP]/d[t] = k_1 [Input][P_n]-k_2[IP][Bst]$

$d[Bst]/d[t] = -k_2[IP][Bst]+k_3 [Ip\text{-}Bst]$

$d[IP\text{-}Bst]/d[t] = k_2 [IP][Bst]-k_3 [IP\text{-}Bst]$

$d[W_{s1}]/d[t] = k_3 [IP\text{-}Bst]$

$d[Us]/d[t] = k_3 [IP\text{-}Bst]-k_4 [Us][Rp]$

$d[Rp]/d[t] = -k_4 [Us][Rp]$

$d[Signal]/d[t] = k_4 [Us][Rp]-k_7 [Rc][Signal]$

$d[Rp\text{-}Us]/d[t] = k_4 [Us][Rp]-k_5 [Rp\text{-}Us][\lambda\ Exo]$

$d[\lambda Exo]/d[t] = -k_5 [Rp\text{-}Us][[\lambda\ Exo]+k_6 [Rp\text{-}Us\text{-}\lambda]$

$d[Rp\text{-}Us\text{-}\lambda]/d[t] = k_5 [Rp\text{-}Us][\lambda\ Exo]-k_6 [Rp\text{-}Us\text{-}\lambda]$

$d[W_{s2}]/d[t] = k_6 [Rp\text{-}Us\text{-}\lambda]$

$d[Rc]/d[t] = k_6 [Rp\text{-}Us\text{-}\lambda]-k_7 [Rc][Signal]$

Rate constants

$k_1 = 2.3x10^4 M^{-1}S^{-1}$
$k_2 = 1.625x10^7 M^{-1}S^{-1}$
$k_3 = 0.167 S^{-1}$
$k_4 = 3.75x10^4 M^{-1}S^{-1}$
$k_5 = 9.97x10^7 M^{-1}S^{-1}$
$k_6 = 0.75 S^{-1}$
$k_7 = 1.0x10^4 M^{-1}S^{-1}$

Referring to the magnitudes of *k* values for various reactions as reported in the referenced literature, we defined the respective ranges of variation for k_1 to k_7 within the system. In this dynamic model, we employed the standard deviation (σ) between the experimental and fitted curves as a measurement criterion. As the σ value stabilizes, it allows us to identify the unknown *k* values that provide the best fit to the experiment results. During the fitting process, we assume that the enzymes operate under saturated conditions, with substrates tightly interacting with the enzymes.

The fitted rate constants are situated within reasonable limits. For instance, Bst exhibits a binding rate constant of 2×10^5 $M^{-1}S^{-1}$, λ Exo demonstrates a binding rate constant of 1.16×10^6 $M^{-1}S^{-1}$, and the hybridization rate constant for single strands is 6.48×10^5 $M^{-1}S^{-1}$.[1-6] The deviation between the fitted rate constant (*k*) and the reference values from the literature is within a single order of magnitude, affirming its effectiveness. This difference may stem from

the impact of actual reaction conditions or variations in the model construction.

To align with the unit of species concentration in the simulation, we converted all DNA and enzyme concentrations. Enzyme activity concentrations (U/mL) were transformed into apparent concentrations (nM), for instance, Bst at 160 U/mL corresponds to 0.16 nM. Furthermore, the fluorescence signal is positively correlated with the concentration of free fluorescent strands, and the DNA components can be converted using the following formula: $(F_t-F_{min})/(F_{max}-F_{min})\times[Signal]$, where F_t represents real-time fluorescence intensity, F_{max} and F_{min} respectively denote the fluorescence intensity of free fluorescent strands and hybridized fluorescent strands. [Signal] represents the concentration of Signal strand.

3. Machine learning method:

1) Data Preprocessing and Feature Engineering

The model input undergoes a rigorous preprocessing pipeline to ensure numerical stability and maximize feature importance. Initially, raw data are transposed and aligned to ensure that samples are represented as rows and kinetic features as columns. We then applied feature scaling using Standard Scaling, also known as Z-score normalization, to transform the features to have a mean of 0 and a standard deviation of 1. This step is critical for preventing features with larger scales from dominating the model during training. While the framework includes a pipeline for Principal Component Analysis (PCA) to reduce feature redundancy, the final model utilized the full feature set to maintain maximum information retention from the rich pulse-like signals.

2) Model Architecture: Random Forest Classifier

The primary model employed in this study is a Random Forest Classifier, an ensemble learning method that operates by constructing a multitude of decision trees during the training phase. We utilized an ensemble strategy based on Bootstrap Aggregating (Bagging) to train 100 individual decision trees (n_estimators = 100) on distinct subsets of the data. To ensure robustness, the final classification is determined by a majority vote across all trees; this approach significantly mitigates the risk of overfitting inherent in single decision trees and enhances the model's ability to generalize to unseen data.

3) Training and Testing Methodology

The reliability of the model was verified using a standard "Hold-out" validation approach, where the dataset was partitioned into two independent subsets: 70% for training and 30% for testing, utilizing a fixed random state to ensure the reproducibility of the results. The model was fitted using the training set (X_{train}, Y_{train}) to capture the non-linear relationships between

the features and the labels. Subsequently, the model's performance was evaluated on the 30% "unseen" test set using four comprehensive metrics: accuracy to assess overall correctness; precision and recall, calculated using weighted averaging to account for potential class imbalances; the F1-score, which serves as the harmonic mean of precision and recall; and confusion matrices, utilized as visual tools to identify specific misclassifications between labels.

Supporting Figure

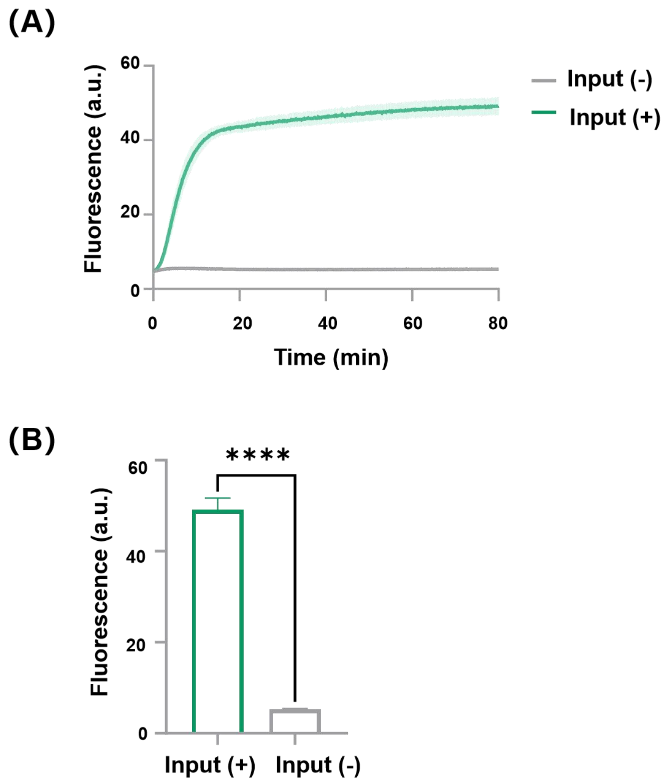

Figure S1. Verification of Us production in the concentration converter. (A) Fluorescence curves showing that the presence of input initiates concentration conversion and reporter activation. (B) Data confirming effective concentration conversion only in the presence of input. All experiments were conducted with 50 nM Input, 50 nM P_n, 200 nM Rp, and 160 U/mL Bst in Thermopol buffer at 25°C. Data are presented as mean ± SD (n = 3 independent experiments).

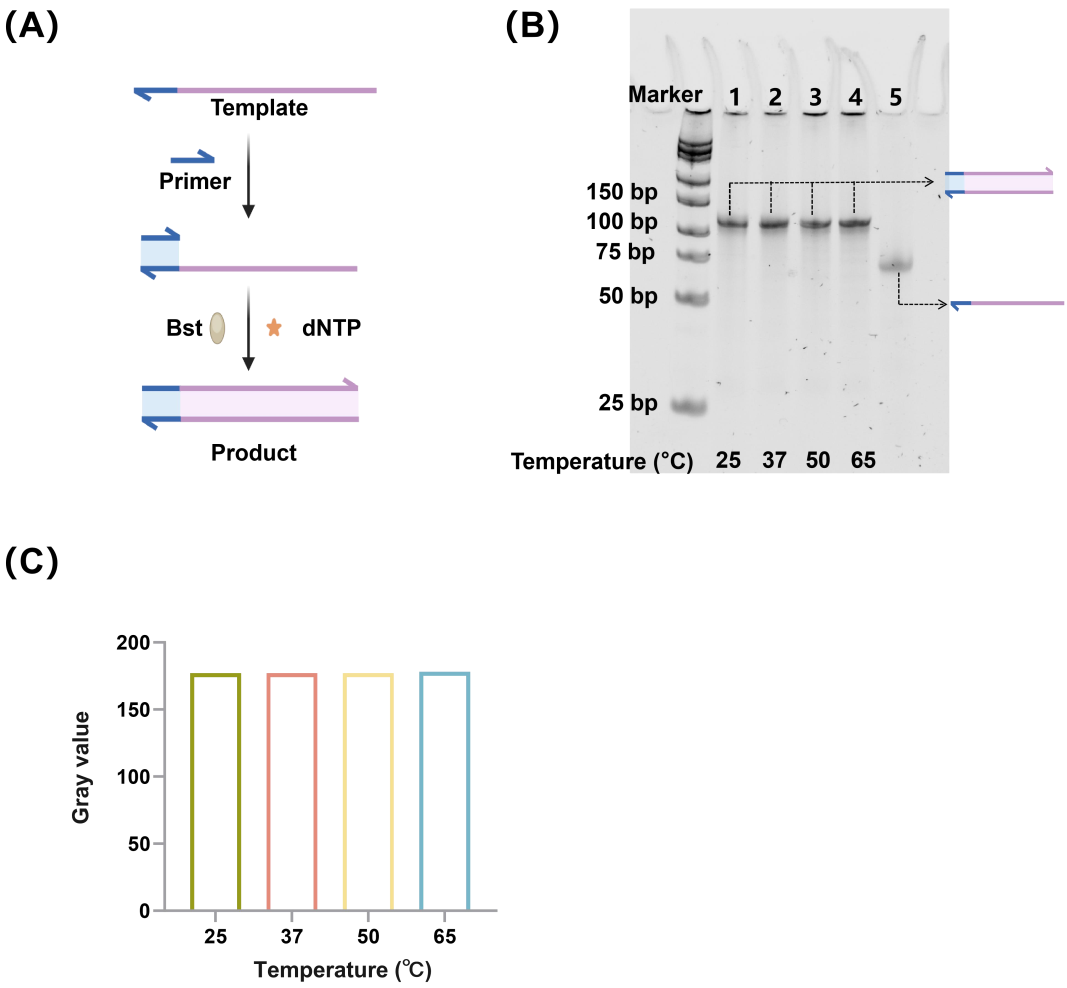

Figure S2. Temperature dependence of Bst polymerase–mediated primer extension. (A) Schematic of the Bst polymerase–mediated primer extension assay. (B) DNA template and primer (each 200 nM) were incubated with Bst polymerase (160 U mL^{-1}) for 60 min at the indicated temperatures, followed by enzyme inactivation at 80°C. Lanes 1-4 correspond to 25, 37, 55, and 65°C. (B) Densitometric quantification (gray value) of the product band intensity as a function of temperature.

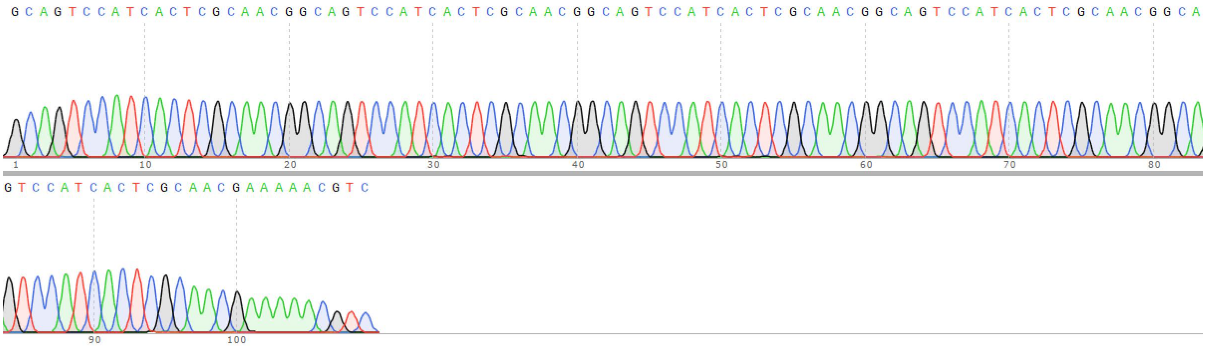

Figure S3. Sequencing validation of the Bst extension products. The base calls across the analyzed region match the designed sequence, with no detectable mixed bases or systematic substitutions.

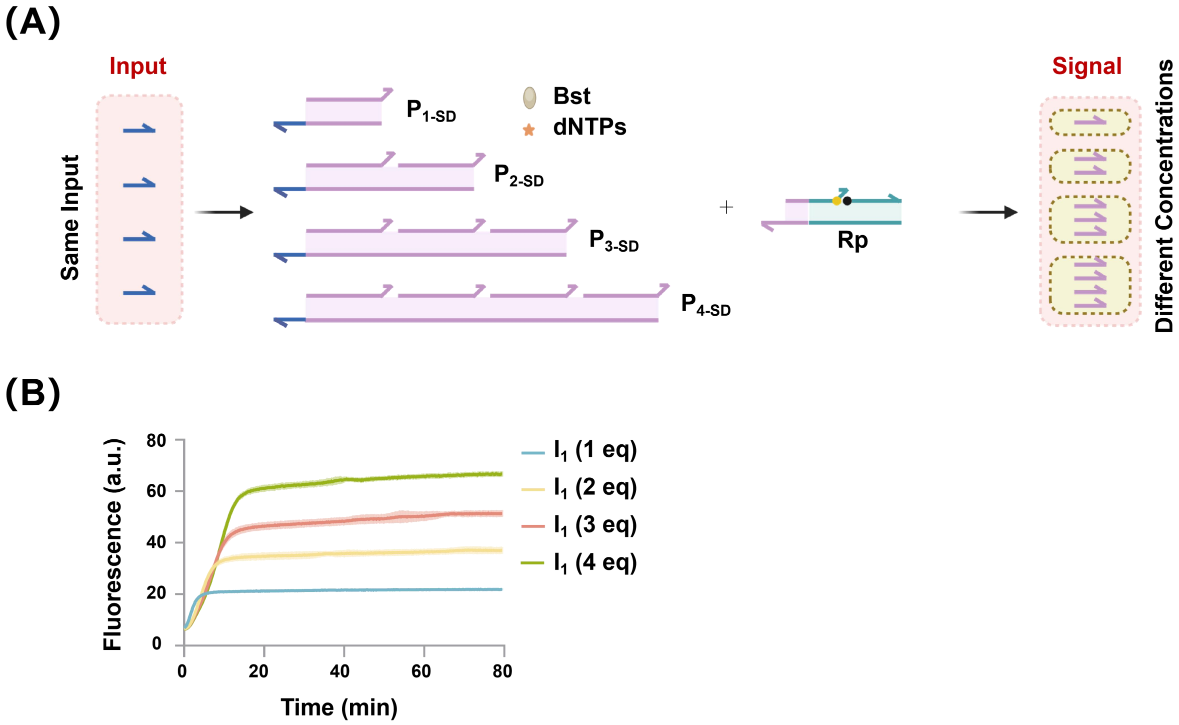

Figure S4. Tunable conversion of the same input into different concentration. (A) Schematic illustration of the conversion of the same input into different concentrations. (B) Fluorescence curves. The same input can be programmed to generate 1-4 eq of Us. All experiments were conducted with 50 nM I_1, 50 nM $P_{n\text{-SD}}$, 200 nM Rp, and 160 U/mL Bst in Thermopol buffer at 25°C. Data are presented as mean ± SD (n = 3 independent experiments).

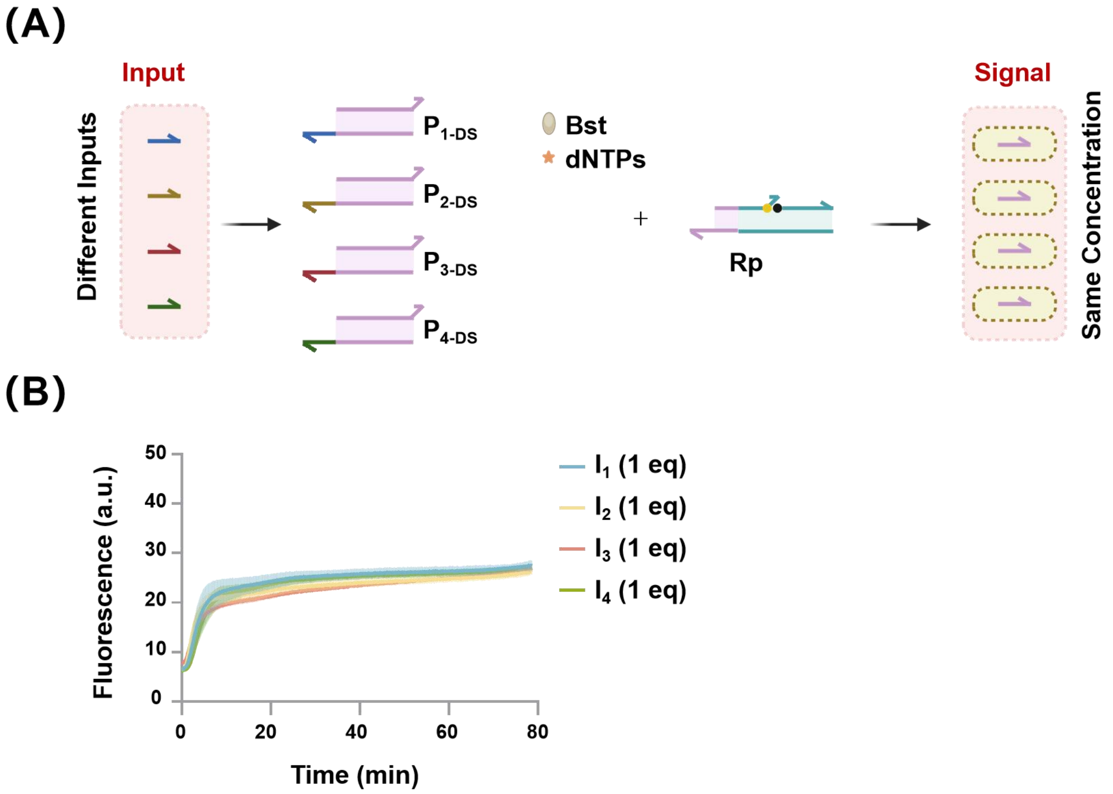

Figure S5. Conversion of different inputs into the same concentration. (A) Schematic illustration of the conversion of the different inputs into same concentration. (B) Fluorescence curves. Different inputs can be programmed to generate 1 eq of Us. All experiments were conducted with 50 nM I_n, 50 nM $P_{n\text{-DS}}$, 200 nM Rp, and 160 U/mL Bst in Thermopol buffer at 25°C. Data are presented as mean ± SD (n = 3 independent experiments).

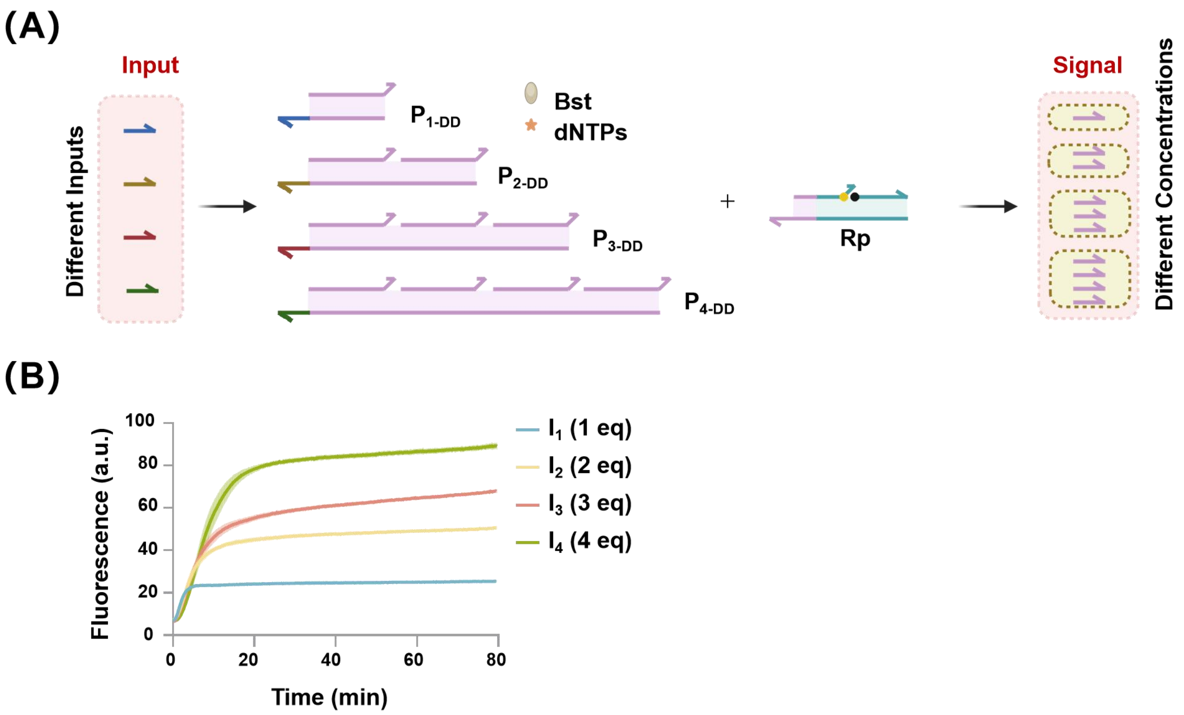

Figure S6. Conversion of different inputs into different Us concentrations. (A) Schematic illustration of the conversion of the different inputs into different concentrations. (B) Fluorescence curves. Different inputs can be programmed to generate 1-4 eq of Us. All experiments were conducted with 50 nM I_n, 50 nM $P_{n\text{-DD}}$, 200 nM Rp, and 160 U/mL Bst in Thermopol buffer at 25°C. Data are presented as mean ± SD (n = 3 independent experiments).

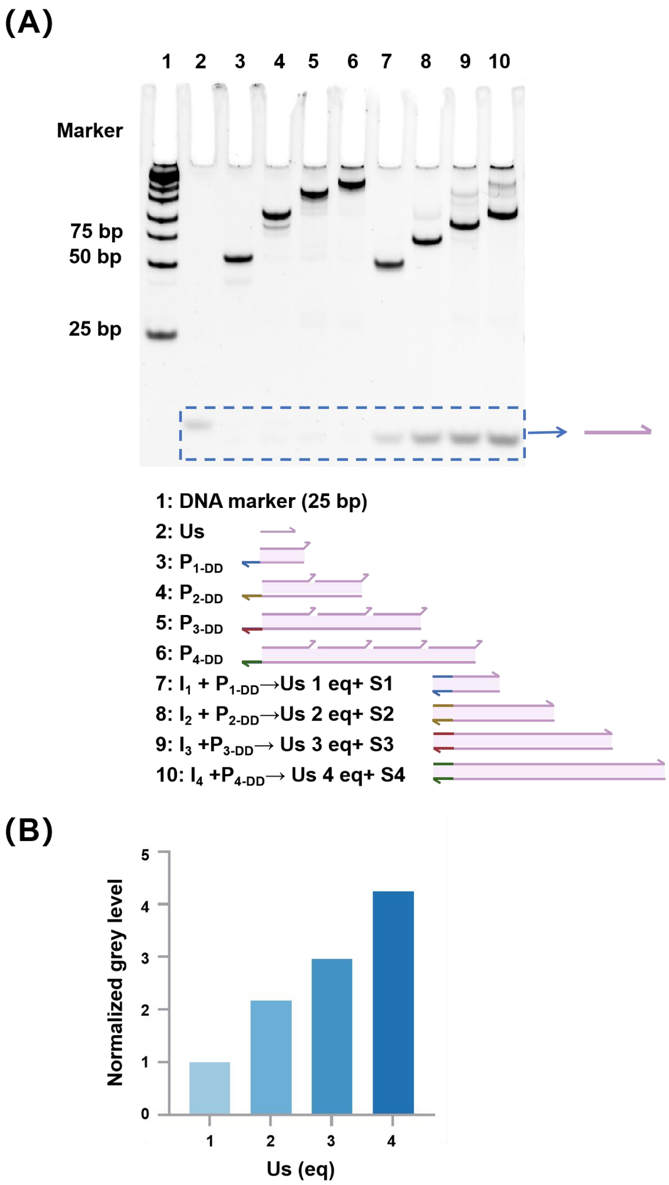

Figure S7. Gel electrophoresis validation of the concentration converter. (A) Native PAGE analysis (20%) of probes and reaction products. Lanes 7–10: products formed after incubation of input (I_n) with the corresponding probes, yielding 1–4 eq of Us. The dashed box highlights released Us, while the upper bands correspond to probe–input complexes after polymerase extension. (B) Grey level of panel A. The lanes 7–10 shows a positive correlation between band intensity and programmed Us concentration. All experiments were conducted with 200 nM I_n, 200 nM $P_{n\text{-DD}}$, 160 U/mL Bst in Thermopol buffer at 25°C.

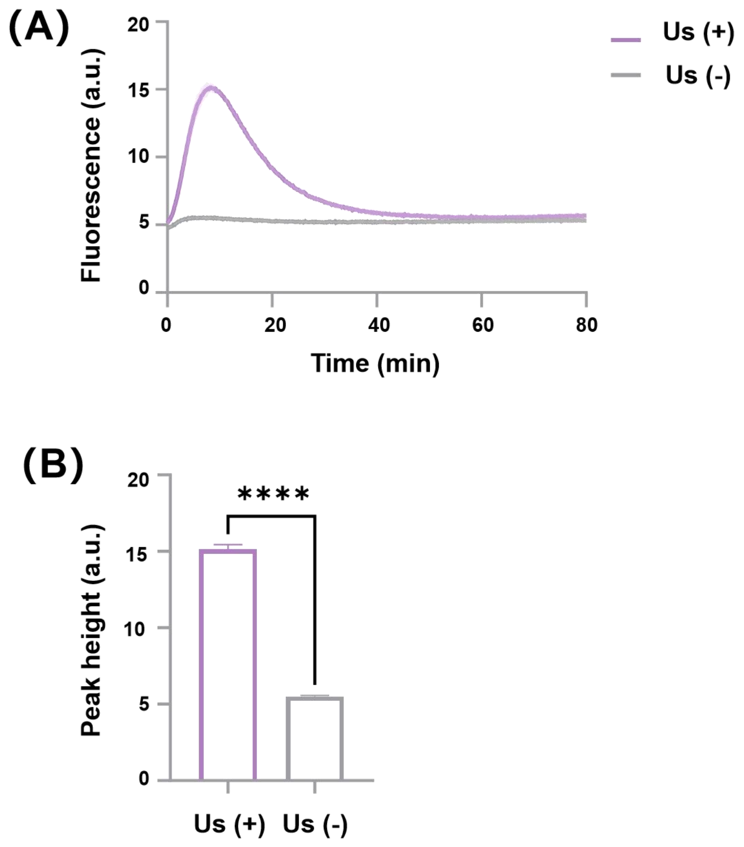

Figure S8. Verification of the temporal decoder. (A) Fluorescence curves of the temporal decoder. The presence of Us produces a pulse-like trajectory, while its absence results in a flat baseline. (B) Peak height of panel A. All experiments were conducted with 50 nM Us, 200 nM Rp, and 5.55 U/mL λ Exo in 1 × Lambda reaction buffer at 25°C. Data are presented as mean ± SD (n = 3 independent experiments).

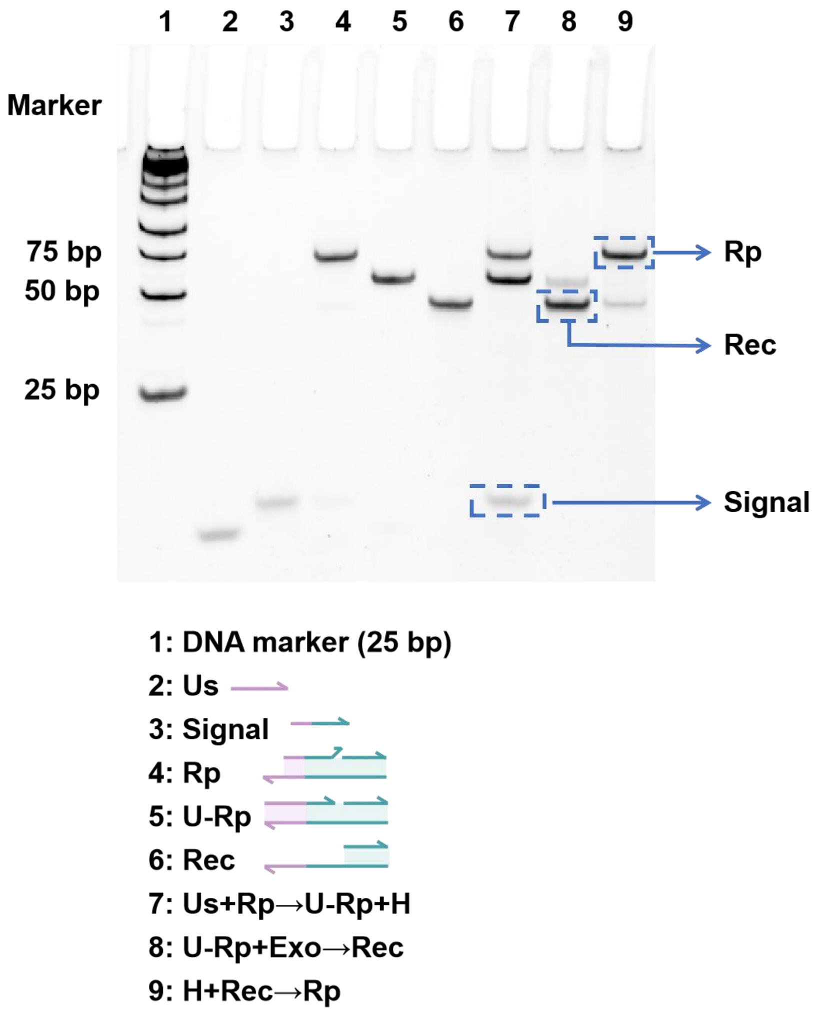

Figure S9. Gel electrophoresis validation of the temporal decoder. Lane 7 confirms H release upon probe activation, lane 8 demonstrates Rec formation after λ Exo digestion, and lane 9 shows Rp regeneration from Rec and Signal. All experiments were conducted with 200 nM Us, 200 nM Signal, 200 nM Rp, 200 nM U-Rp, 200 nM Rec, and 5.55 U/mL λ Exo in 1× Lambda reaction buffer at 25°C.

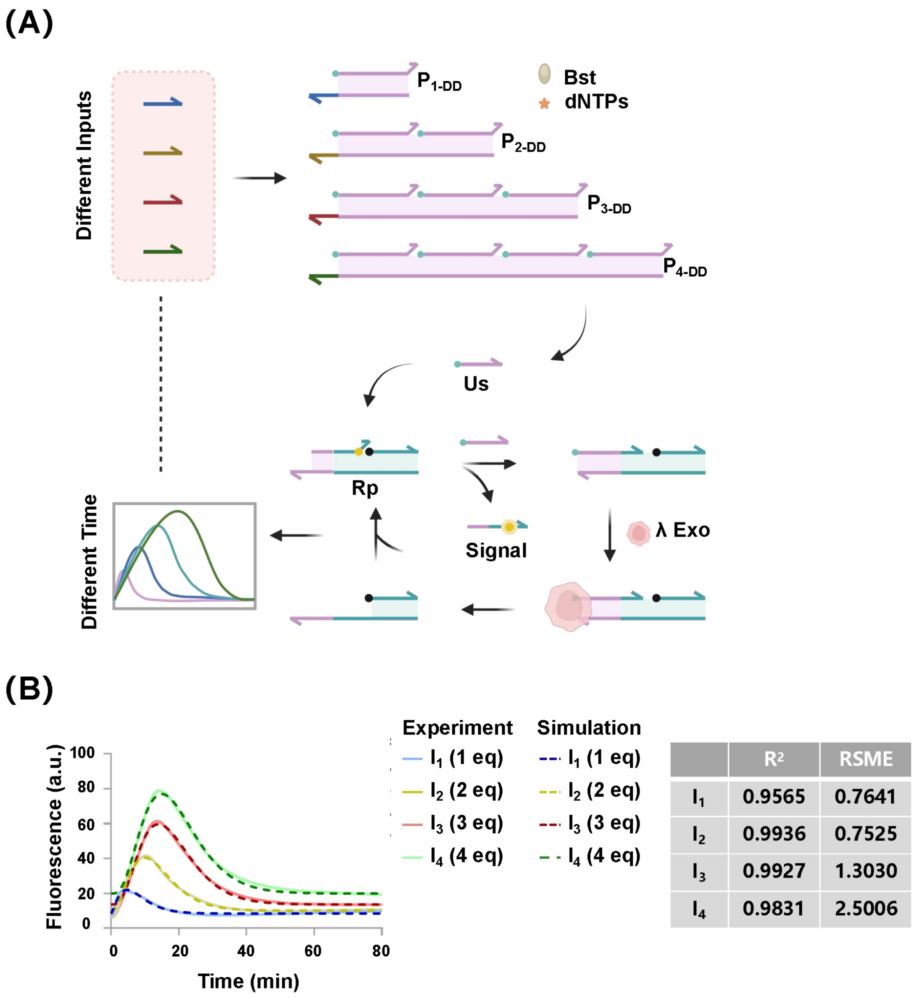

	R^2	RSME
I_1	0.9565	0.7641
I_2	0.9936	0.7525
I_3	0.9927	1.3030
I_4	0.9831	2.5006

Figure S10. Different temporal signatures programmed from the same inputs. (A) Schematic illustration of the conversion of same inputs into different temporal signatures. (B) Experimental fluorescence trajectories (solid) overlaid with ODE simulations (dashed) for the conditions in (A), with R^2 and RMSE reported for each input. The complete reaction equations and rate constants are provided in the Supporting Notes. All experiments were conducted with 50 nM I_1, 50 nM $P_{n\text{-SD}}$, 200 nM Rp, 160 U/mL Bst and 5.55 U/mL λ Exo in 1× Lambda reaction buffer at 25°C. Data are presented as mean ± SD (n = 3 independent experiments).

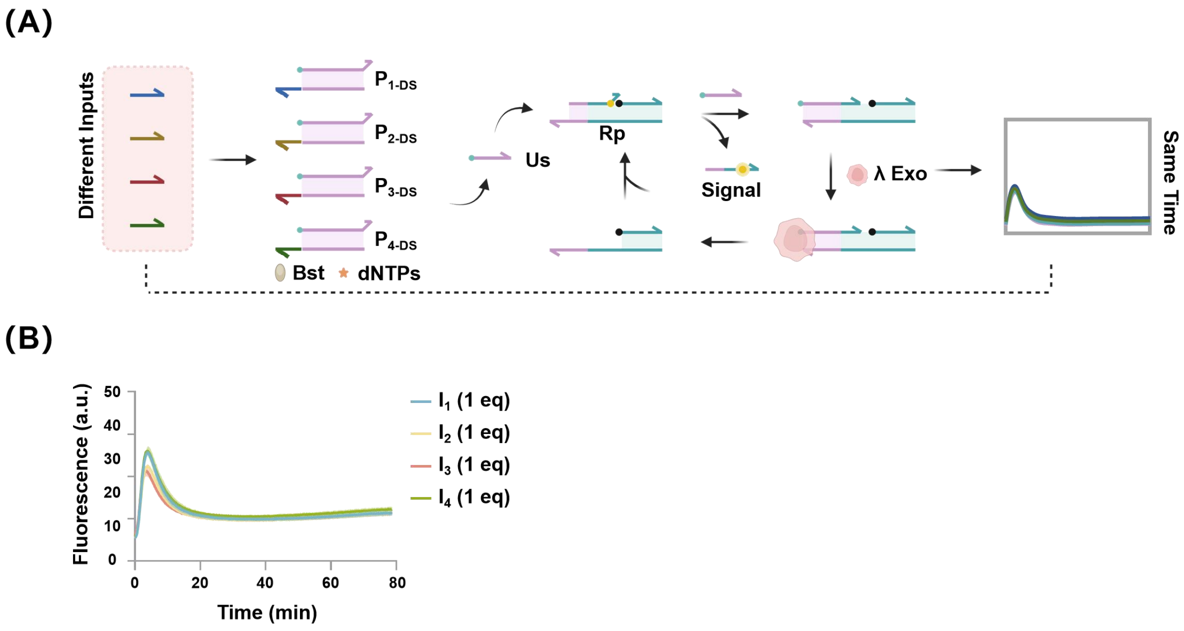

Figure S11. The same temporal signatures programmed from different inputs. (A) Schematic illustration of the conversion of different inputs into same temporal signatures. (B) Fluorescence curves of (A). All experiments were conducted with 50 nM I_n, 50 nM $P_{n\text{-DS}}$, 200 nM Rp, 160 U/mL Bst and 5.55 U/mL λ Exo in 1× Lambda reaction buffer at 25°C. Data are presented as mean ± SD (n = 3 independent experiments).

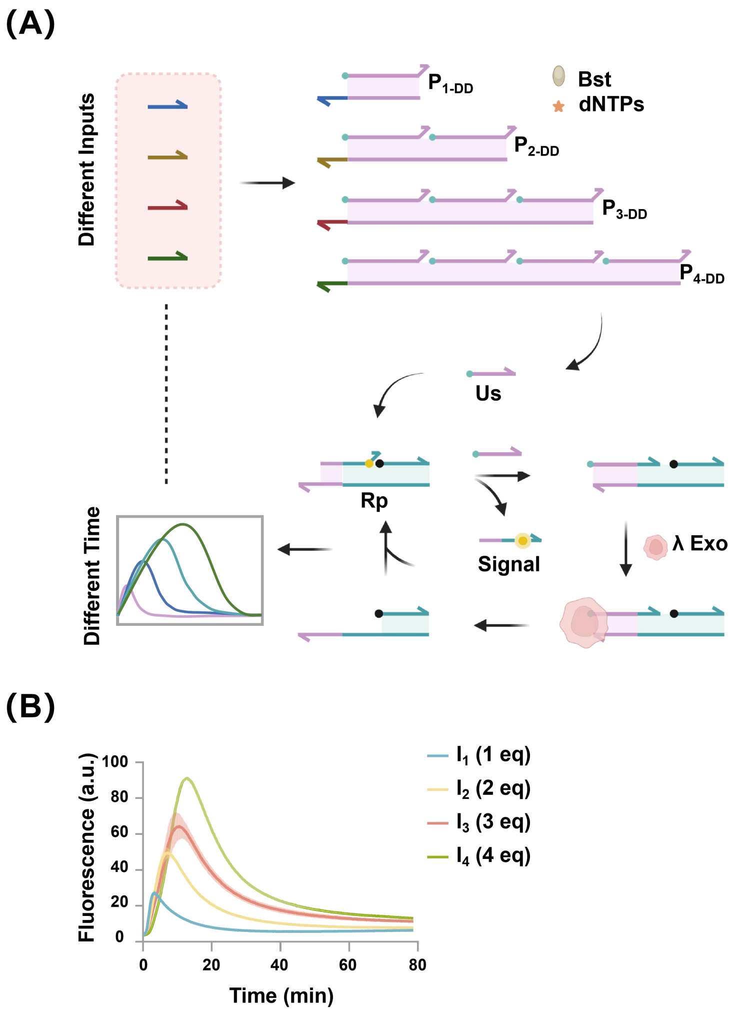

Figure S12. Different temporal signatures programmed from different inputs. (A) Schematic illustration of the conversion of different inputs into different temporal signatures. (B) Fluorescence curves of (A). All experiments were conducted with 50 nM I_n, 50 nM $P_{n\text{-DD}}$, 200 nM Rp, 160 U/mL Bst and 5.55 U/mL λ Exo in 1 × Lambda reaction buffer at 25°C. Data are presented as mean ± SD (n = 3 independent experiments).

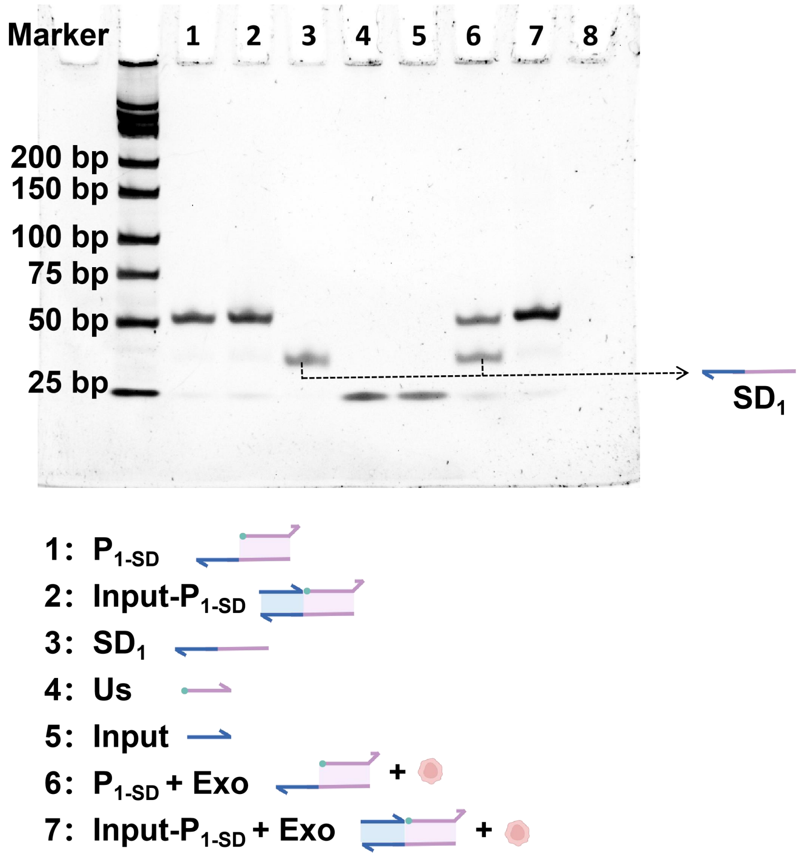

Figure S13. Gel electrophoresis validation of Input-mediated protection of the 5′-phosphorylated recessed-end probe against λ Exo digestion.Lane 6 shows partial probe degradation, whereas lane 7 largely preserves the Input–probe duplex band with markedly reduced degradation products, indicating that Input hybridization shields the recessed 5′-phosphorylated end and suppresses λ Exo access.

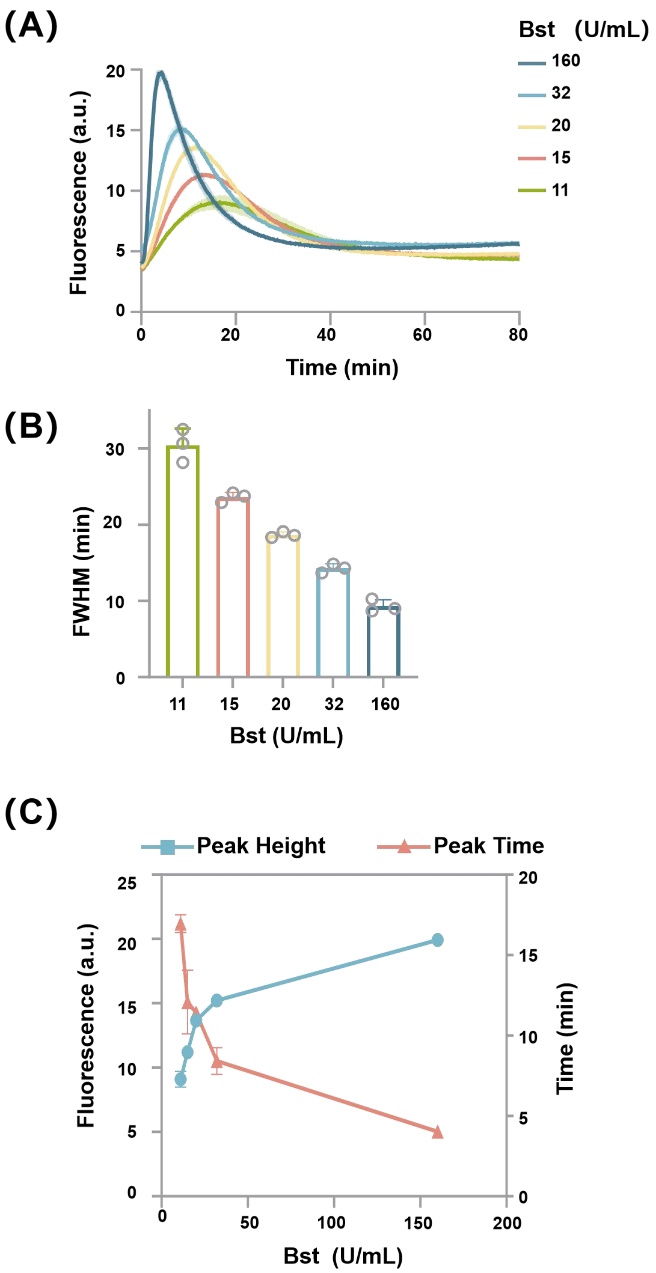

Figure S14. Regulation of temporal signatures by Bst concentration. (A) Fluorescence curves of different Bst concentrations (11–160 U/mL). (B) FWHM extracted in panel A. FWHM decreases monotonically with increasing Bst, indicating shorter signal lifetimes at higher enzyme levels. (C) Peak height and peak time extracted in panel A. With increasing Bst, peak height increases while peak time decreases, showing that higher Bst advances the peak and sharpens the transient. All experiments were conducted with 50 nM I_1, 50 nM $P_{1\text{-SD}}$, 200 nM Rp and 5.55 U/mL λ Exo in 1 × Lambda reaction buffer at 25°C.

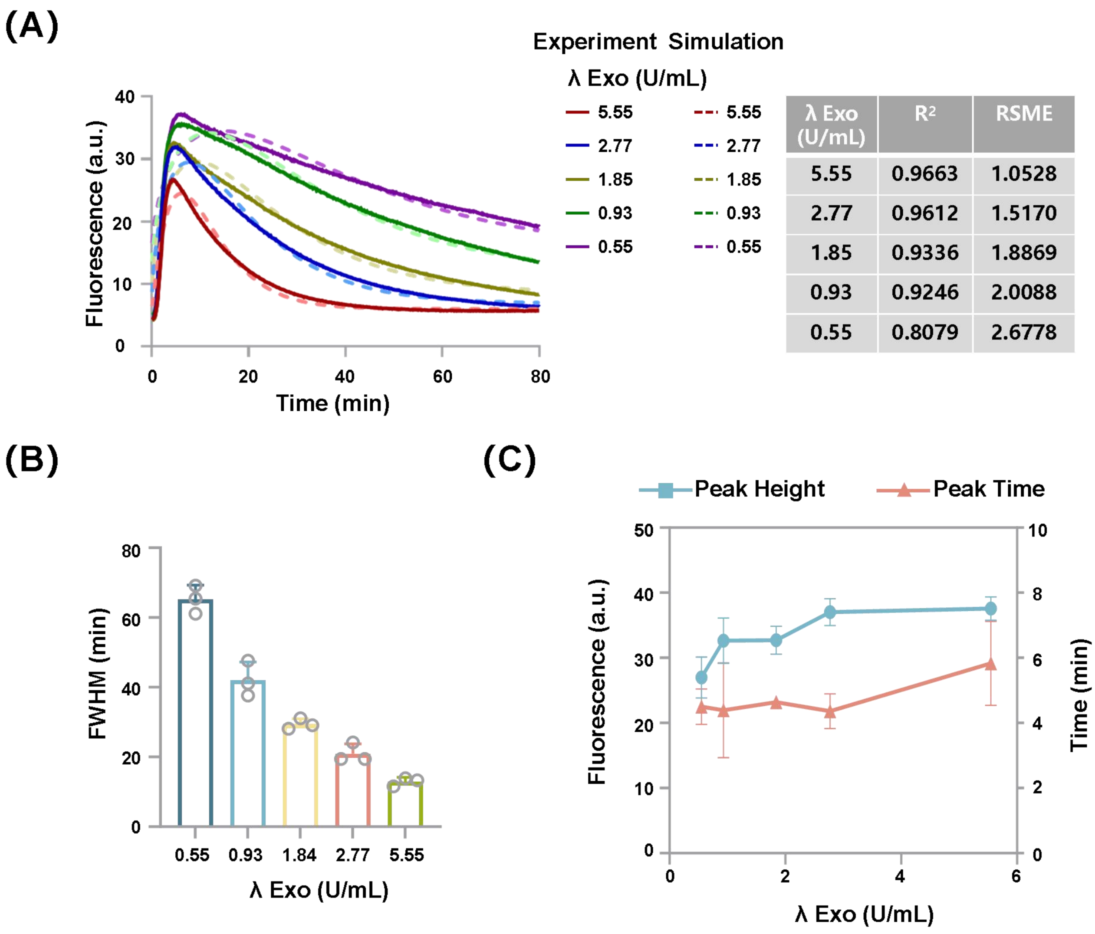

λ Exo (U/mL)	R^2	RSME
5.55	0.9663	1.0528
2.77	0.9612	1.5170
1.85	0.9336	1.8869
0.93	0.9246	2.0088
0.55	0.8079	2.6778

Figure S15. Regulation of temporal signatures by λ Exo concentration. (A) Experimental fluorescence trajectories at varying λ Exo concentrations (0.55-5.55 U/mL) overlaid with ODE simulations (dashed), with R^2 and RMSE reported for each condition. The complete reaction equations and rate constants are provided in the Supporting Notes. (B) FWHM extracted in panel A. FWHM decreases monotonically with increasing λ Exo. (C) Peak time and peak height extracted in panel A. With increasing λ Exo concentration, both peak height and time to peak showed minimal changes. All experiments were conducted with 50 nM I_1, 50 nM $P_{1\text{-SD}}$, 200 nM Rp and 160 U/mL Bst in 1× Lambda reaction buffer at 25°C. Data are presented as mean ± SD (n = 3 independent experiments).

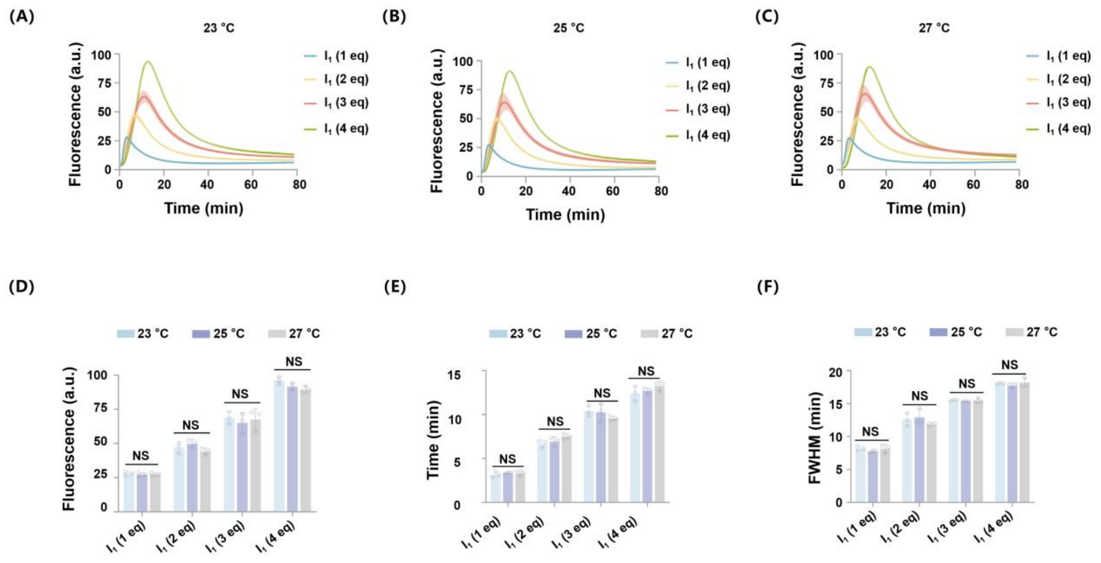

Figure S16. Temperature tolerance of the EDRN (23-27°C). (A) Fluorescence curves of the EDRN measured at 23°C. (B) Fluorescence curves of the EDRN measured at 25°C. (B) Fluorescence curves of the EDRN measured at 27°C. (D) Peak height of the EDRN pulse signals at 23°C, 25°C, and 27°C. (E) Peak time of the EDRN pulse signals at 23°C, 25°C, and 27°C. (F) FWHM of the EDRN pulse signals at 23 °C, 25°C, and 27°C.

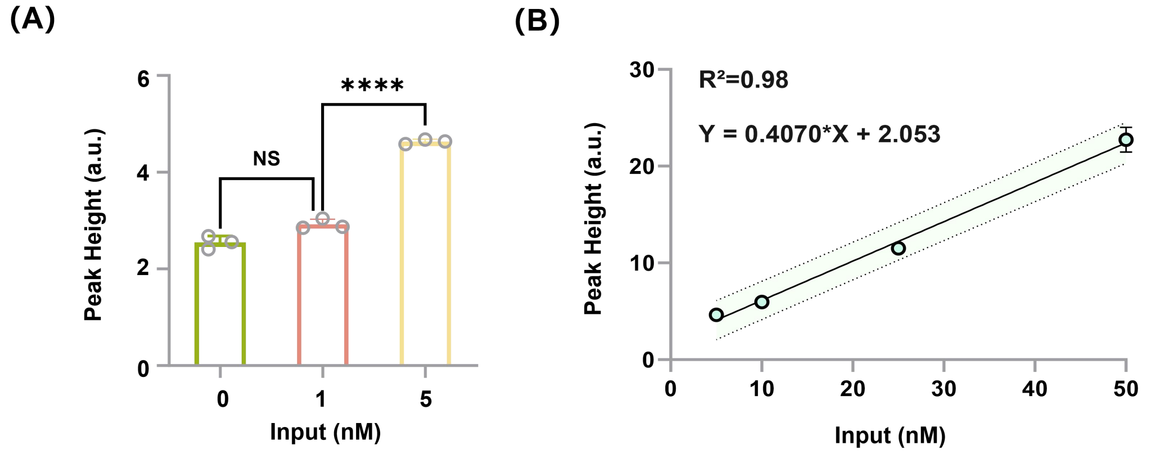

Figure S17. EDRN's native sensitivity and dynamic range. (A) Determination of the intrinsic limit of detection (LOD) of the EDRN module. Peak-height analysis comparing 0, 1, and 5 nM inputs shows no significant difference between 0 and 1 nM and a significant increase at 5 nM (****, $p < 0.0001$).(B) Linear dynamic range of the EDRN module. Peak height scales linearly with input concentration over 5–50 nM (R^2=0.98).

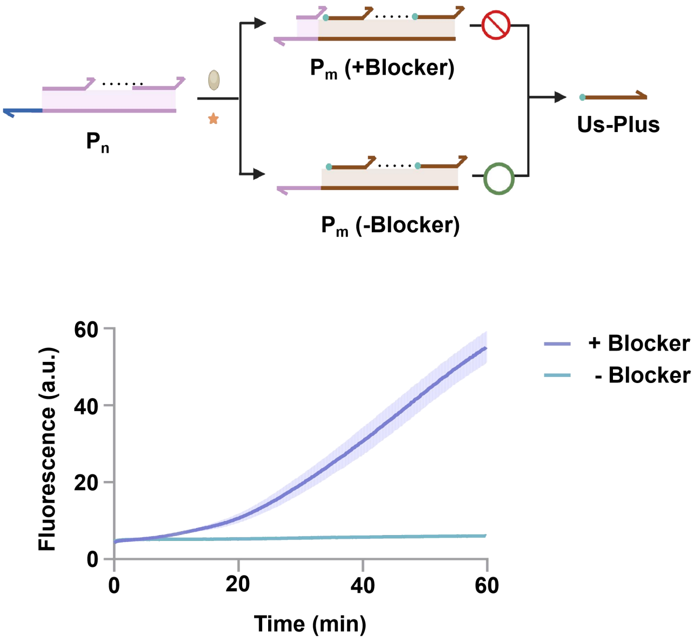

Figure S18. Blocker suppresses cross-talk–induced leakage in the two-layer concentration converter. When the P_m module contains Blocker, cross-talk between the two layers is effectively inhibited, yielding no detectable fluorescence in the absence of input. In contrast, removing the Blocker leads to pronounced input-free signal leakage.

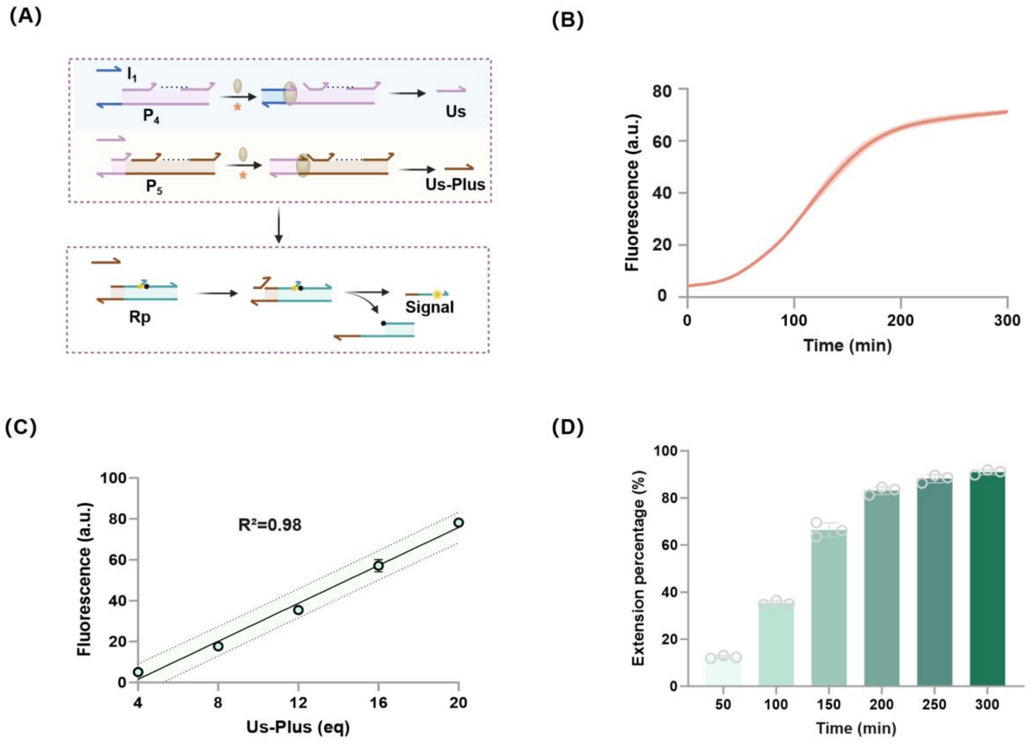

Figure S19. Characterization of the polymerase extension efficiency of the double-layer concentration converter. (A) Reporting the released Us-Plus concentration. (B) Fluorescence kinetics of product formation. Time-course fluorescence measurement (0-300 min) showing monotonic signal accumulation and an approach to plateau, consistent with gradual product buildup in the converter. (C) Calibration of fluorescence versus Us-Plus concentration. (D) Time-resolved quantification of polymerase extension.

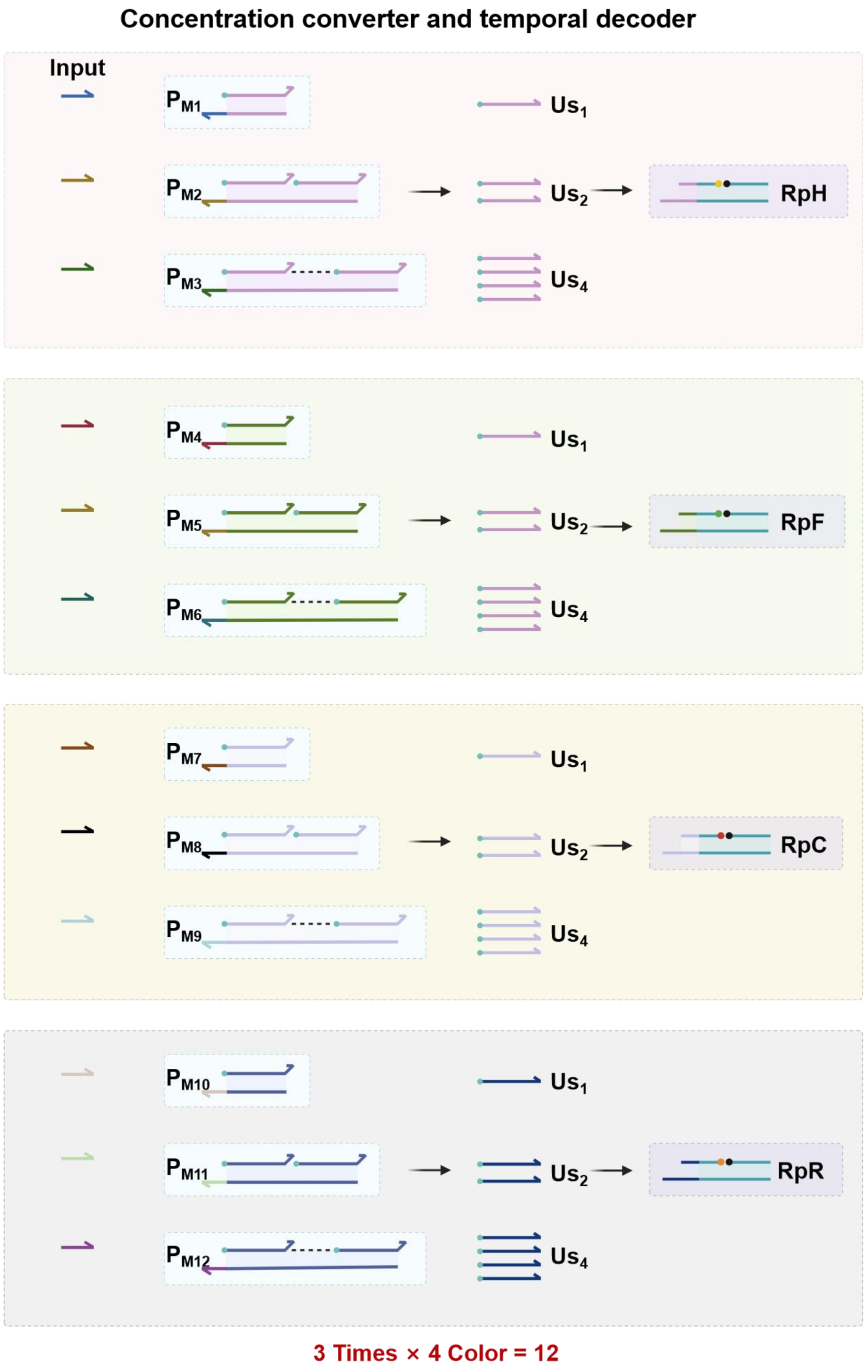

Figure S20. Design details of EDRN for multiplexing detection. Three concentration converters were each paired with four temporal decoders with different dyes. Taking one temporal decoder as an example, three different targets could be detected, as the targets were first converted into different concentrations of Us and subsequently decoded into temporal outputs. In this manner, this strategy allows for the simultaneous identification of at least 12 targets.

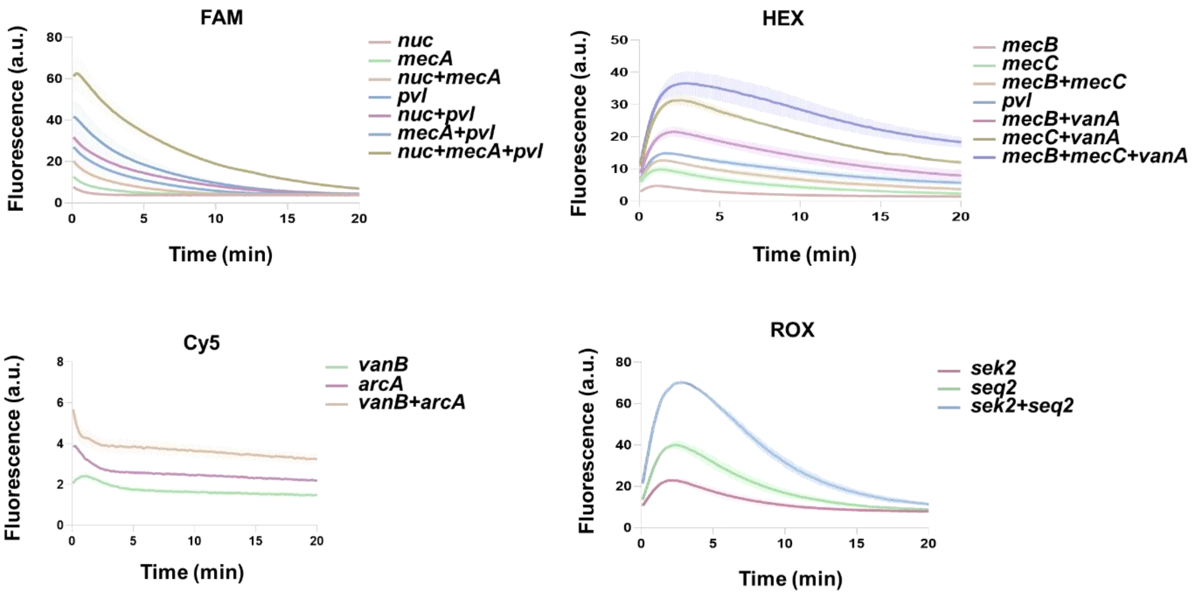

Figure S21. Multiplexed detection of synthetic targets by EDRN. Fluorescence curves of single targets and representative combinations shown by fluorescence channels. All experiments were conducted with 20-140 nM Us, 140 nM Rp, and 8.33 U/mL λ Exo in 1× Lambda reaction buffer at 25°C. Data are presented as mean ± SD (n = 3 independent experiments).

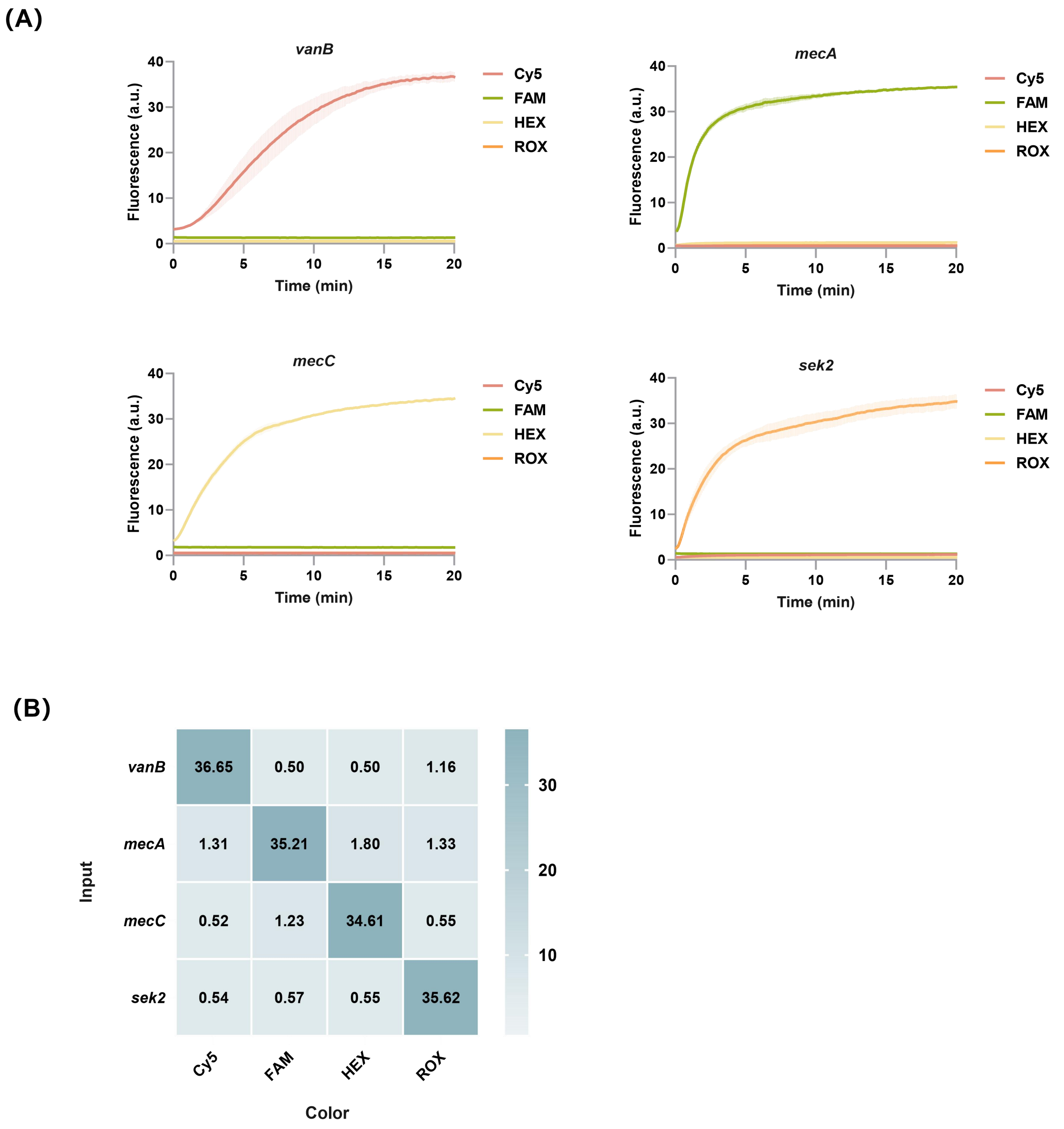

Figure S22. Minimal spectral crosstalk among four fluorescence channels in the multiplex EDRN assay. (A) Fluorescence kinetics of the concentration converter across Cy5, FAM, HEX, and ROX for individual targets (vanB, mecA, mecC, and sek2). Each target activates only its assigned channel, with other channels remaining at baseline. (B) Crosstalk matrix summarizing endpoint fluorescence responses of each target measured across all four channels.

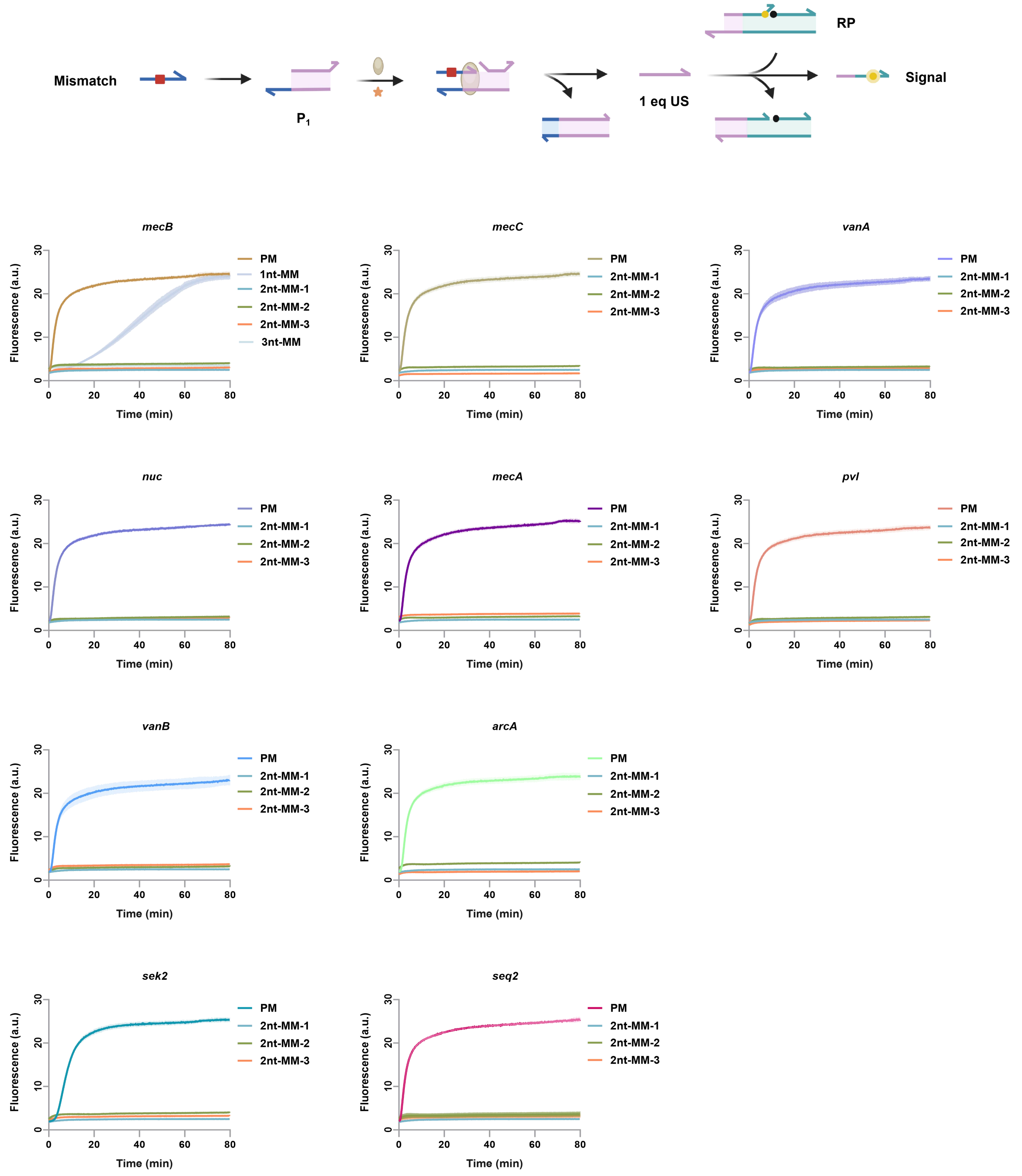

Figure S23. Specificity validation of EDRN against mismatched variants for 10 clinically relevant target sequences. For each target sequence, the corresponding concentration converter probe was used at 1 eq under identical assay conditions. All experiments were conducted with 50 nM Probe, 200 nM Rp, 160 U/mL Bst, and 8.33 U/mL λ Exo in 1× Lambda reaction buffer at 25°C. Data are presented as mean ± SD (n = 3 independent experiments).

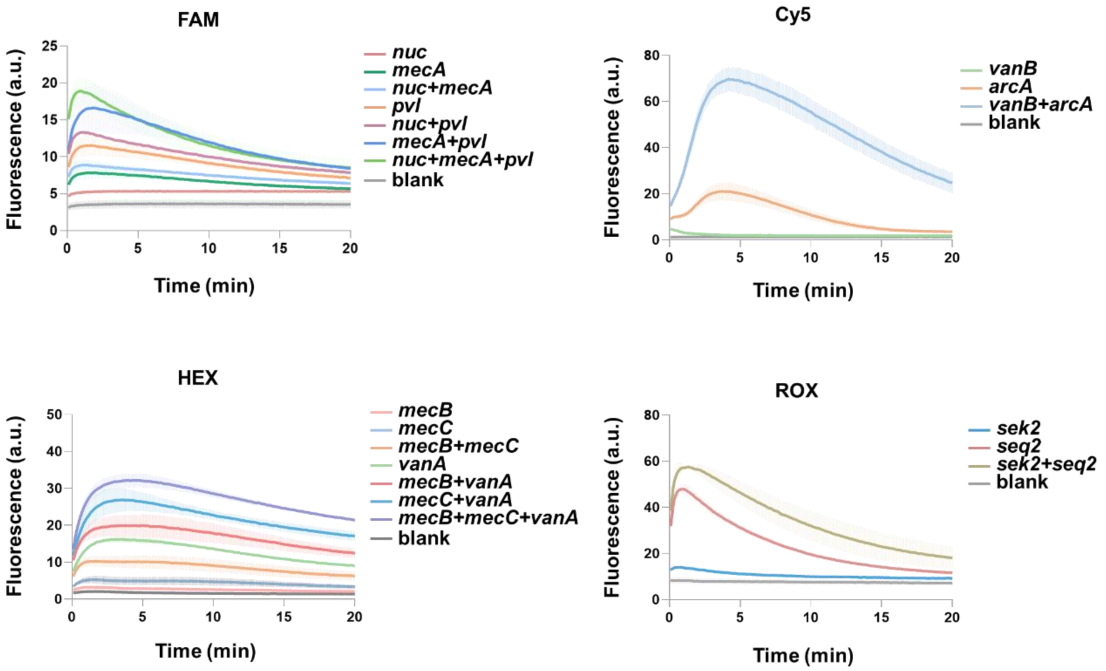

Figure S24. Multiplexed detection of amplified fragments by EDRN genetic targets. Fluorescence curves of single targets and representative combinations shown by fluorescence channels. The data was used for training the machine learning. All experiments were conducted with 20 nM Probe, 140 nM Rp, 160 U/mL Bst, and 8.33 U/mL λ Exo in 1× Lambda reaction buffer at 25°C. Data are presented as mean ± SD (n = 3 independent experiments).

	Accuracy	Recall	F1-score	Precision
FAM	0.95	0.95	0.96	0.95
HEX	1.00	1.00	1.00	1.00
Cy5	1.00	1.00	1.00	1.00
ROX	0.98	0.98	0.98	0.98

Figure S25. Performance metrics of machine learning (accuracy, recall, precision, and F1-score) for FAM, HEX, Cy5, and ROX channels. All channels showed excellent performance, confirming the robustness of detection system.

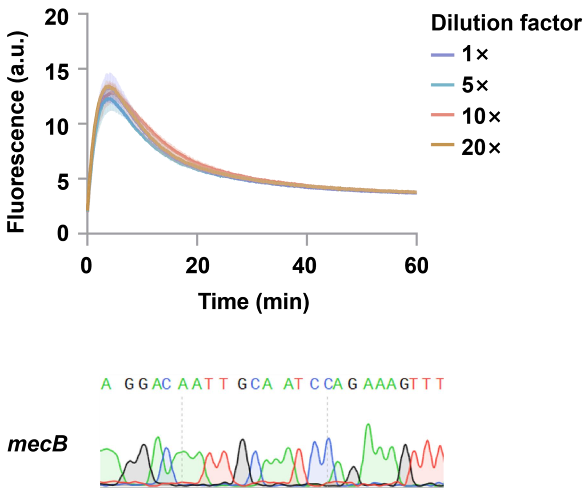

Figure S26. Validation using diluted clinical samples to assess the effect of initial template copy number on EDRN readout. A clinical sample confirmed to be positive for the *mecB* gene by sequencing was diluted by 1×, 5×, 10×, and 25× before PCR amplification, and the corresponding PCR products were then subjected to the EDRN reaction. The resulting fluorescence curves showed similar trends and signal intensities across all tested dilution conditions.

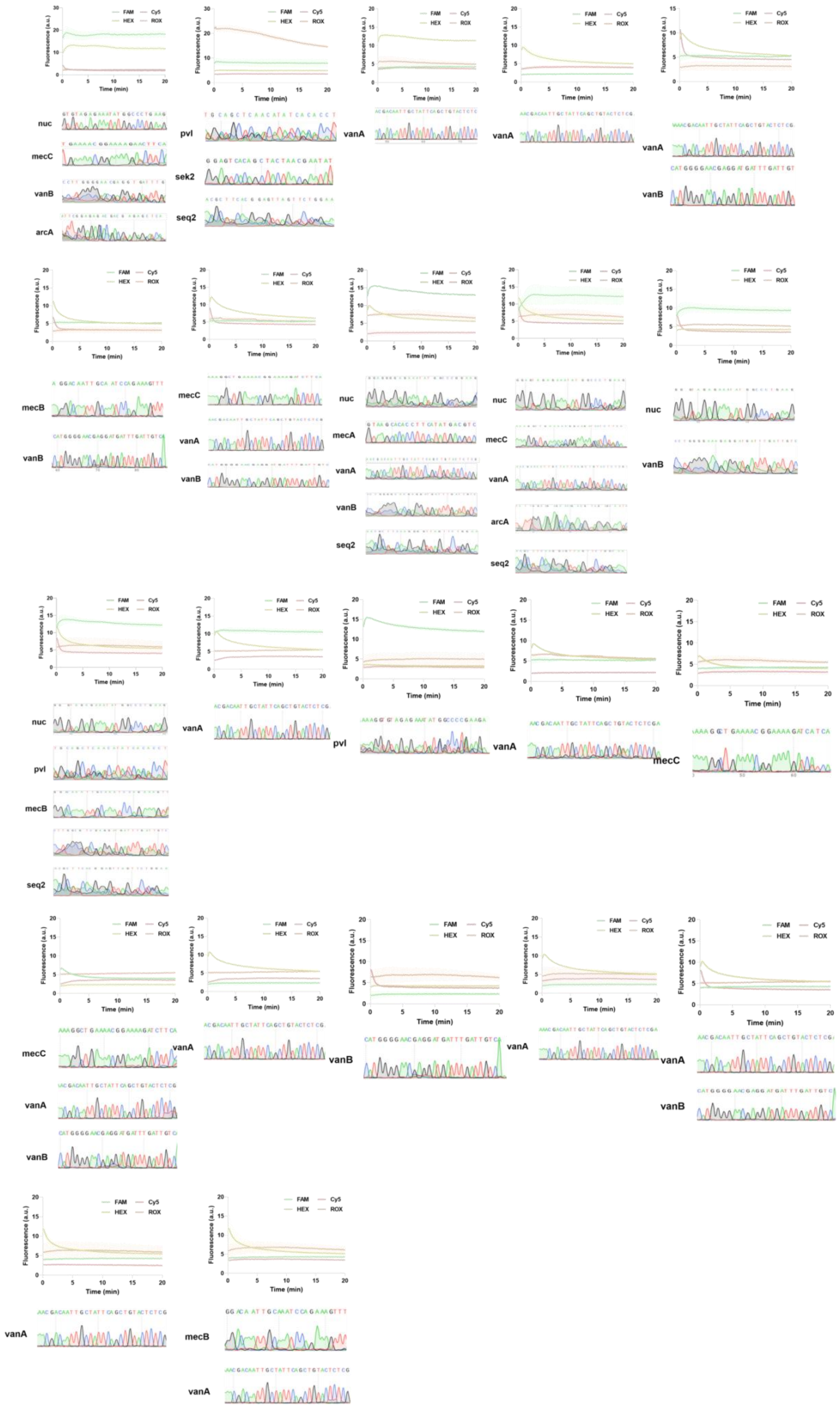
FAM
HEX
Cy5
ROX
Fluorescence (a.u.)
Time (min)
nuc
mecC
vanB
arcA
pvl
sek2
seq2
vanA
mecB
mecA

Figure S27. Fluorescence kinetic curve of positive clinical samples associated with the sequencing results. All experiments were conducted with 20 nM Probe, 140 nM Rp, 160 U/mL Bst, and 8.33 U/mL λ Exo in 1 × Lambda reaction buffer at 25 °C. Data are presented as mean ± SD (n = 3 independent experiments).

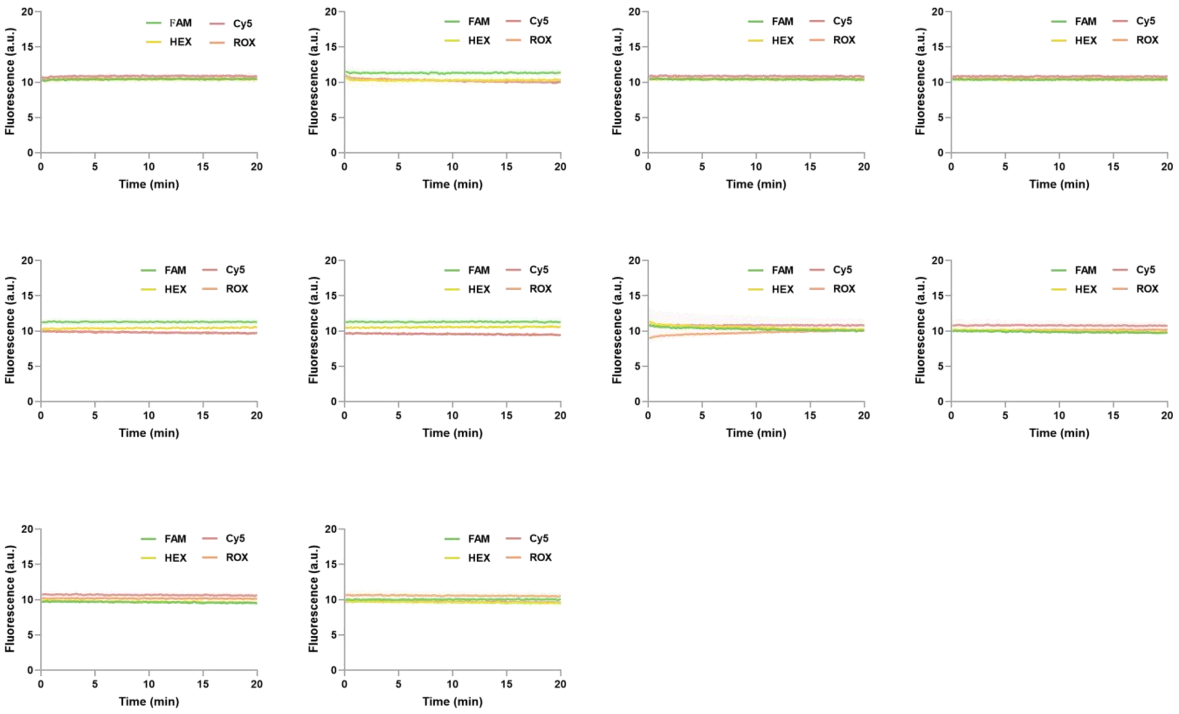

Figure S28. Fluorescence kinetic curve of negative clinical samples. All experiments were conducted with 20 nM Probe, 140 nM Rp, 160 U/mL Bst, and 8.33 U/mL λ Exo in 1× Lambda reaction buffer at 25°C. Data are presented as mean ± SD (n = 3 independent experiments).

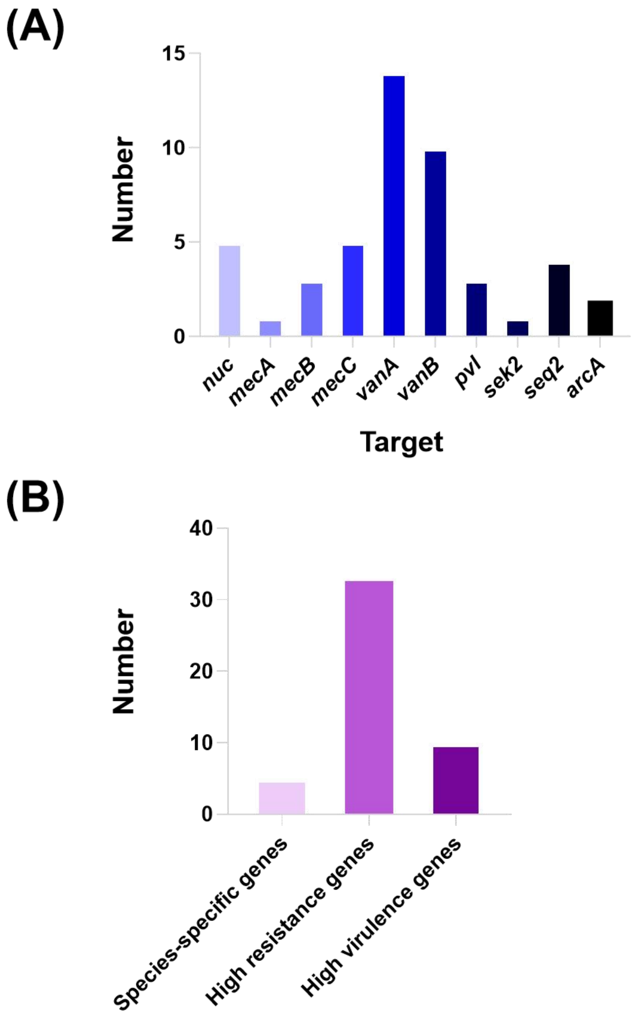

Figure S29. Summary of MRSA gene detection results in clinical samples. (A) Distribution of detected gene types across 22 infected clinical samples. Bars indicate the number of positive cases for each gene, including species-specific (*nuc*), resistance-associated (*mecA*, *mecB*, *mecC*, *vanA*, *vanB*), and virulence-related genes (*pvl*, *sek2*, *seq2*, *arcA*). (B) Functional classification of detected genes, showing dominance of high-resistance genes, followed by species-specific and virulence markers.

References

[1] Y. Liu, S. Fu, J. Liu and X. Su, "A DNA-Based Dissipation System that Synchronizes Multiple Fuels," *Chemistry* 29 (2023): e202301156.
[2] N. Li, Y. Zhao, Y. Liu, Z. Yin, R. Liu, L. Zhang, L. Ma, X. Dai, D. Zhou and X. Su, "Self-resetting molecular probes for nucleic acids detection enabled by fuel dissipative systems," *Nano Today* 41 (2021): 101308.
[3] J. Liu, Y. Liu, L. Zhang, S. Fur and X. Su, "Ultra-specific fluorescence detection of DNA modifying enzymes by dissipation system," *Biosensors & bioelectronics* 215 (2022): 114561 .
[4] J. G. Wetmur, N. Davidson, "Kinetics of renaturation of DNA," *Journal of molecular biology vol* 31 (1968): 349-70.
[5] L. A. Frederico, T. A. Kunkel and B. R. Shaw, "A sensitive genetic assay for the detection of cytosine deamination: determination of rate constants and the activation energy," *Biochemistry* 29 (1990), 2532–2537.
[6] J. C. Hsieh, S. Zinnen and P. Modrich, "Kinetic mechanism of the DNA-dependent DNA polymerase activity of human immunodeficiency virus reverse transcriptase." *The Journal of biological chemistry vol* 268 (1993): 24607-24613.
